# Antisense oligonucleotides treatment uncovers differences in the modulation of dysregulated intracellular pathways in Spinal Muscular Atrophy motoneurons

**DOI:** 10.1038/s41419-026-09105-1

**Published:** 2026-07-13

**Authors:** Maria P. Miralles, Isabel Gimenez-Fernandez, Maria Beltran, Alba Sansa, Laura Martínez-España, Jordi Calderó, Manuel Portero-Otín, Rosa M. Soler, Ana Garcera

**Affiliations:** 1 Neuronal Signaling Unit, Experimental Medicine Department, Universitat de Lleida, Institut de Recerca Biomèdica de Lleida-Fundació Dr Pifarré (IRBLleida), Lleida, Spain; 2 Patologia Neuromuscular Experimental, Experimental Medicine Department, Universitat de Lleida, Institut de Recerca Biomèdica de Lleida-Fundació Dr Pifarré (IRBLleida), Lleida, Spain; 3 Metabolic Pathophysiology Research Group, Experimental Medicine Department, Universitat de Lleida, Institut de Recerca Biomèdica de Lleida-Fundació Dr Pifarré (IRBLleida), Lleida, Spain; 4 Present Address: The Board of Governors Regenerative Medicine Institute, Cedars-Sinai Medical Center, Los Angeles, CA USA

**Keywords:** Neurodegeneration, Mechanisms of disease

## Abstract

Spinal Muscular Atrophy (SMA) is a neuromuscular genetic disorder resulting from the mutation or deletion of the *Survival Motor Neuron 1* (*SMN1*) gene and the reduction of the Survival Motor Neuron (SMN) protein. As a result, SMN level in SMA depends on the almost identical copy gene *SMN2*, which produces a small amount of functional SMN due to a silent mutation in exon 7. SMN deficiency critically impairs spinal cord motoneuron (MN) function, causing progressive degeneration. Advances in SMA therapeutics have significantly improved clinical management and prognosis. However, therapeutic outcomes vary among SMA patients, resulting in broad heterogeneity in phenotypes and clinical trajectories; consequently, more focused investigation on the underlying disease mechanisms is essential. One of the FDA-approved treatments is nusinersen, an antisense oligonucleotide (ASO) designed to enhance *SMN2* exon 7 splicing and increase SMN protein. The present study used non-SMA and SMA MNs differentiated from human induced Pluripotent Stem Cells (hiPSCs) to analyze the effect of a nusinersen-like ASO treatment on intracellular pathways altered in SMA MNs. ASO treatment efficiently increased SMN, prevented MN degeneration, and decreased apoptotic markers in SMA MNs. Furthermore, treatment increased Gemin3 protein and the NF-κB members, IKKβ and RelA. Nevertheless, ASO did not revert alterations of the autophagy markers LC3-II and p62/SQSTM1, and the calpain activation product α-fodrin 145/150 kDa. Our observations indicate that nusinersen-like ASO treatment might be insufficient to counteract the full spectrum of intracellular alterations occurring in SMN-reduced MNs. Therefore, supplementary compounds targeting these unrecovered pathways might supply additional protective effects on degenerating MNs.

## Introduction

Spinal Muscular Atrophy (SMA) is a monogenic neuromuscular disease caused by a reduction in the Survival Motor Neuron (SMN) protein. SMN is predominantly codified by the *SMN1* gene; when *SMN1* gene is deleted or mutated, SMN level depends exclusively on the *SMN2* gene [1–3], making this paralogous gene *SMN2* the main modifier of SMA severity. A single nucleotide modification in *SMN2* exon 7 causes the majority of transcripts to be alternatively spliced, leading to lower levels of the functional full-length SMN [4, 5]. SMA patients have variable *SMN2* gene copy numbers, ranging from one to eight copies. In most cases, a higher number of *SMN2* copies is associated with a less severe phenotype [6, 7]; additional genetic modifiers of SMA have also been identified [8, 9].

SMN is a ubiquitous and essential protein for cellular physiology. SMN housekeeping functions include the assembly of spliceosomal small nuclear ribonucleoproteins (snRNPs) and the formation of complexes due to SMN’s intrinsic binding capacity with a large number of proteins [10–12]. Below a specific threshold, it disrupts numerous molecular and cellular processes such as miRNA biogenesis, p53-related RNA damage, apoptosis, proteasomal degradation, autophagy, transport of RNA granules along the axons, and endocytosis [11, 13–16]. Although phenotypes outside the central nervous system have been reported in SMA patients and mouse models [17, 18], spinal cord MNs and neuromuscular junction are primarily affected in SMA [19, 20]. Despite this broad spectrum of compromised cellular processes, the cause(s) underlying MN dysfunction remain to be identified.

Splicing correction or increasing expression of *SMN2* have been proposed as the main strategies for disease-modifying therapies in SMA [21]. Recent advances in increasing functional SMN protein have shown remarkable success in treating SMA patients. Currently, three SMA therapies are available: intrathecal-delivered anti-sense oligonucleotide (ASO) (Nusinersen) [22, 23], orally administrated small molecules *SMN2* splicing modifiers (Risdiplam) [24], or intravenous adeno-associated virus-based gene delivery of *SMN1* (Onasemnogene abeparvovec) [25]. Nusinersen was the first drug approved, and more than 11,000 people have been treated worldwide [26]. Despite the beneficial effects of these treatments in the majority of SMA patients, the clinical response varies and new SMA phenotypes are emerging [27]. Improving treatment outcomes requires identifying the molecular mechanisms reversed by therapy. This understanding is crucial for guiding the development of novel strategies aimed at optimizing existing therapeutic approaches [28]. The main objective of the present work was to determine the effect of the ASO treatment on altered intracellular mechanisms using an in vitro model of SMA MNs differentiated from human induced Pluripotent Stem Cells (hiPSCs). SMN reduction causes dysregulation in intracellular pathways related to MN function. For instance, protein members of apoptosis, autophagy, and cell survival pathways are altered in SMA cells [29–35]. Moreover, the inhibition of the calcium-dependent protease calpain increases Smn protein and ameliorates SMA phenotype in mice [36, 37] and SMN protein acts as a regulator of the SMN-complex members Gemin3 and Gemin5 [38, 39]. These proteins and pathways compromised in SMA are candidates for correction by treatment. In this context, we explored the effect of a nusinersen-like ASO (*SMN*-ASO) on some of the pathways modified in SMA cells. We established an in vitro model of 20-day differentiated human MNs from non-affected control, SMA-carrier and SMA patient-derived hiPSC. *SMN*-ASO treatment increased SMN protein, reduced apoptosis features, and prevented neurite degeneration in SMA differentiated MNs. Moreover, the protein level of Gemin3, a member of the SMN-complex, was increased in ASO-treated SMA MNs. This observation, combined with the upregulation of RelA and IKKβ proteins, suggests that downregulation of Gemin3 and NF-κB pathway are involved in the degeneration process observed in non-treated SMA cells. Nevertheless, ASO treatment did not decrease LC3-II and p62/SQSTM1 autophagy markers, and the calpain-specific fodrin breakdown product. Our findings highlight the need to further dissect the pathways dysregulated in SMA MNs, with particular attention to observing their modulation by treatment and defining their contribution to disease mechanisms and progression. Such knowledge could inform the rational design of combined therapies and provide new avenues to counteract MN degeneration in SMA.

## Results

### SMN protein level in differentiated human MNs derived from iPSCs

**Fig. 1:**
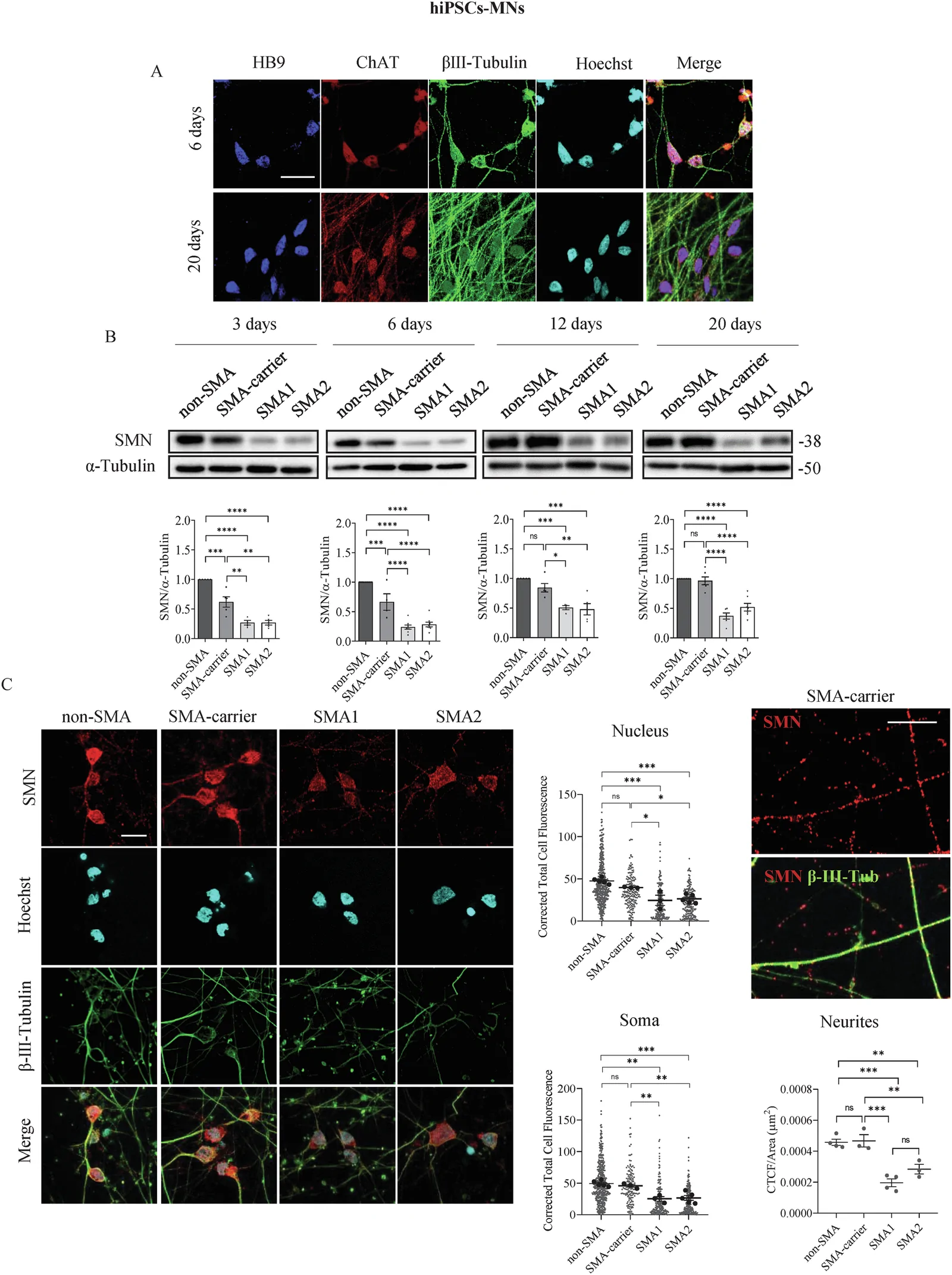
Analysis of SMN protein level in differentiated human MNs derived from iPSCs. **A** Representative immunofluorescence images of 6-day and 20-day differentiated human MNs showing HB9 (blue), ChAT (red), and β-III-Tubulin (green). Hoechst (cyan) staining was used to identify cell nucleus. Scale bar 30 μm. **B** Protein extracts of 3-day, 6-day, 12-day, and 20-day differentiated non-SMA, SMA-carrier, SMA1 and SMA2 MNs were submitted to western blot using anti-SMN antibody. Membranes were reprobed using anti-α-tubulin antibody, used as a loading control. Graph values represent the expression of SMN versus α-tubulin relative to the non-SMA condition (normalized to 1) and correspond to the quantification of at least 4 independent hiPSCs differentiation cycles ± SEM. **C** Representative immunofluorescence images of 20-day differentiated human non-SMA, SMA-carrier, SMA1 and SMA2 MNs using anti-SMN antibody (red), anti-β-III-Tubulin antibody (green), and Hoechst staining (cyan) to identify MN nucleus. Graph values represent the mean of relative SMN fluorescence (Corrected total cell fluorescence, CTCF) measured in the cell nucleus, soma, and neurites (CTFC/Area µm^2^), corresponding to the quantification of at least 25 cells per condition from 4 independent hiPSCs differentiation cycles ± SEM. Scale bar, 15 μm (left) and 10 μm (right). Asterisks indicate significant differences using one-way Anova with Tukey’s multiple comparisons post-test (*p < 0.05, **p < 0.01, ***p < 0.001, ****p < 0.0001, no significant (ns) *p* > 0.05).

To investigate the effect of *SMN*-ASO treatment in cultured SMA MNs, hiPSCs from two SMA patients (SMA1 and SMA2), one SMA carrier (SMA-carrier), and one non-affected control (non-SMA) were differentiated to MNs (see “Materials and methods” and Supplementary Fig. 1). After 6 days and 20 days of differentiation, the MNs markers HB9 and ChAT as well as β-III-tubulin were expressed by these cells (~90–95% of the cells were HB9+ChAT+β-III-tubulin positive), suggesting that hiPSCs were efficiently differentiated to MNs. Using β-III-tubulin immunodetection, it was clearly visible that differentiating neurons extended long processes progressively over time (Fig. 1A). The sustained growth of these processes suggests network maturation throughout the culture period.

To evaluate SMN level in control and SMA cells, we performed a time-course experiment collecting cell lysates after 3, 6, 12, and 20 days of MN differentiation (3 days, 6 days, 12 days, and 20 days, respectively). Protein extracts were submitted to western blot analysis using an anti-SMN antibody. SMA-carrier condition exhibited a significant reduction of SMN protein after 3 d (0.62 ± 0.086, *p* = 0.0006) and 6 d (0.665 ± 0.139, *p* = 0.0002), compared to the non-SMA condition. In contrast, the SMN protein level in SMA-carrier samples (12 days, 0.844 ± 0.071, *p* = 0.3359; 20 days, 0.965 ± 0.065, *p* = 0.9580) was comparable to non-SMA after longer periods (Fig. 1B). Furthermore, SMN was reduced from the initial periods of differentiation until the end of the experiment in SMA1 (3 days, 0.2675 ± 0.04, *p* < 0.0001 and *p* = 0.0020; 6 days, 0.2388 ± 0.035, *p* < 0.0001; 12 days, 0.5075 ± 0.034, *p* = 0.0006 and *p* = 0.0138; 20 days, 0.37 ± 0.052, *p* < 0.0001) and SMA2 (3 days, 0.269 ± 0.039, *p* < 0.0001 and *p* = 0.0012; 6 days, 0.282 ± 0.0398, *p* < 0.0001; 12 days, 0.48 ± 0.095, *p* = 0.0002 and *p* = 0.0049; 20 days, 0.52 ± 0.064, *p* < 0.0001) compared to the non-SMA and the SMA-carrier conditions, respectively (Fig. 1B). No significant differences in SMN content were observed between SMA1 and SMA2 MNs.

After 20 days of differentiation, SMA and control cultures were fixed and submitted to immunofluorescence protocol using an anti-SMN antibody. Confocal images revealed that SMN was reduced in nucleus, soma, and neurites of SMA1 (CTCF, nucleus 21.54 ± 6.023, *p* = 0.0005 and *p* = 0.0257; soma 21.19 ± 3.55, *p* = 0.0011 and *p* = 0.0085; neurites 0.000196 ± 0.00003, *p* = 0.0002 and *p* = 0.0003) and SMA2 (CTCF, nucleus 23.19 ± 2.27, *p* = 0.0003 and *p* = 0.0263; soma 22.77 ± 3.83, *p* = 0.0006 and *p* = 0.0068; neurites 0.000284 ± 0.00003, *p* = 0.0063) MNs, compared to non-SMA (CTCF, nucleus 44.58 ± 1.56; soma 45.37 ± 2.08; neurites 0.0004578 ± 0.00002), and SMA-carrier (CTCF, nucleus 36.75 ± 0.42; soma 41.93 ± 2.3; neurites 0.000467 ± 0.00004). SMA-carrier showed no differences in SMN fluorescence (nucleus: *p* = 0.2674; soma: *p* = 0.8796; neurites: *p* = 0.8238), compared to the non-SMA control. Consistent with western blot experiments, immunofluorescence analysis showed no significant variations in SMN level between SMA1 and SMA2 differentiated MNs (Fig. 1C) (Supplementary Fig. 2).

### *SMN*-ASO treatment increases SMN and reduces neurite degeneration in cultured SMA MNs

**Fig. 2:**
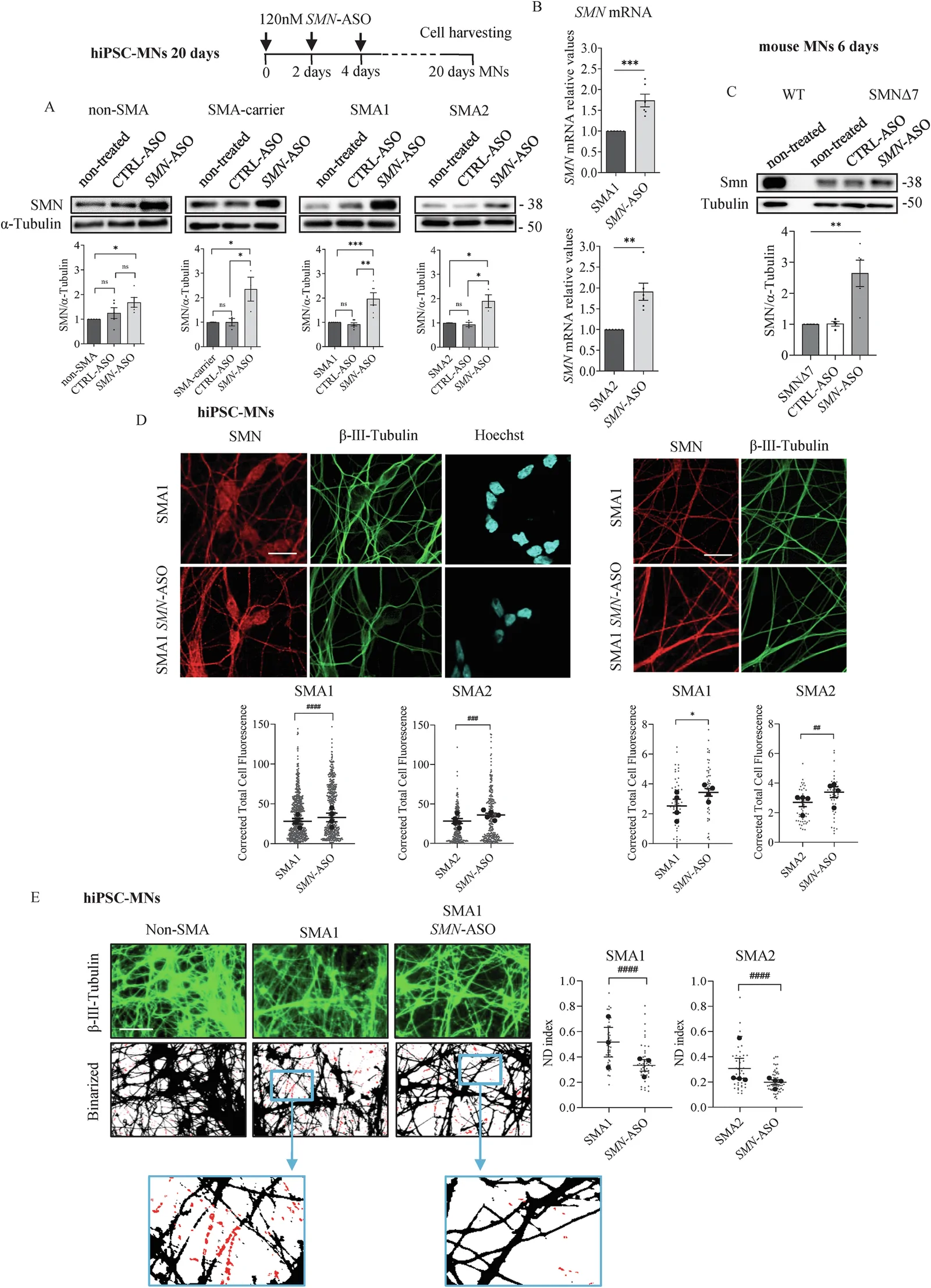
ASOs treatment increases SMN protein and mRNA level and reduces neurite degeneration in SMA differentiated human MNs. **A** Non-SMA, SMA-carrier, SMA1 and SMA2 MNs were treated with 120 nM CTRL-ASO or *SMN*-ASO or left untreated. Total cell lysates from 20-day differentiated MNs were submitted to western blot using an anti-SMN antibody. Membranes were reprobed using an anti-α-tubulin antibody as a loading control. Graph values represent the expression of SMN versus α-tubulin relative to the respective non-treated condition (normalized to 1) and correspond to the quantification of at least 3 independent hiPSCs differentiation cycles ± SEM. Asterisks indicate significant differences using one-way Anova with Tukey’s multiple comparisons post-test (*p < 0.05, **p < 0.01, ***p < 0.001, ns *p* > 0.05). **B** Total mRNA from 20-day differentiated untreated or treated SMA1 and SMA2 MNs was extracted, reverse transcribed to cDNA, and followed by RT-qPCR. *GAPDH* gene was used as control. Graph values are the mean of *SMN* gene expression relative to the respective non-treated SMA condition (normalized to 1) from 3 independent hiPSCs differentiation cycles ± SEM. Asterisks indicate significant differences using Student *t* test (**p < 0.01, ***p < 0.001). **C** WT and SMN∆7 cultured mouse MNs were treated with 120 nM CTRL-ASO or *SMN*-ASO, or left untreated. Protein extracts were collected after 6 days and western blot was performed using an anti-SMN antibody. Membranes were reprobed using anti-α-tubulin antibody. Graph values represent the expression of Smn versus α-tubulin relative to the non-treated SMN∆7 condition (normalized to 1) and correspond to the quantification of at least 3 independent experiments ± SEM. Asterisks indicate significant differences using one-way Anova with Tukey’s multiple comparisons post-test (**p < 0.01). **D** Representative immunofluorescence images of 20-day differentiated non-treated or 120 nM *SMN*-ASO-treated SMA1 MNs using anti-SMN antibody (red) and anti-β-III-Tubulin antibody (green). Hoechst staining (cyan) was used to identify cell nucleus. Graph values represent the mean of relative SMN fluorescence (Corrected Total Cell Fluorescence, CTCF) measured in the cell soma (left) and neurites (right), and correspond to the quantification of at least 30 cells per condition from 4 independent hiPSCs differentiation cycles ± SEM. Scale bar, 15 μm. Symbols indicate differences using Student *t* test (*p < 0.05) or Mann-Whitney test (^##^*p* < 0.01, ^###^*p* < 0.001, ^####^*p* < 0.0001). **E** Representative immunofluorescence images of 20-day differentiated untreated and treated SMA1 MNs using anti-β-III-Tubulin antibody. Blue magnification shows degenerated neurites (red). Scale bar, 50 μm. Graph values represent the area of degenerated neurites versus the total area occupied by β-III-Tubulin (Neurite Degeneration (ND) Index) and correspond to the quantification of 10 images per condition from at least 3 independent hiPSCs differentiation cycles ± SEM. Symbols indicate differences using Mann-Whitney test (^####^*p* < 0.0001).

To investigate the effect of *SMN*-ASO treatment on SMN protein level in SMA MNs, non-SMA and SMA1 hiPSCs were submitted to the differentiation protocol and treated with *SMN*-ASO and CTRL-ASO (see “Materials and methods”). Several doses of ASOs were tested and western blot results indicated that 120 nM *SMN*-ASO was the maximum effective concentration to increase SMN protein level in SMA MNs (see timeline of treatment protocol and western blot analysis in Supplementary Fig. 3). Therefore, control and SMA differentiated MNs were treated with 120 nM *SMN*-ASO or 120 nM CTRL-ASO following the treatment protocol described in Materials and Methods (Supplementary Fig. 3). After 20 days, western blot experiments showed that *SMN*-ASO induced a significant SMN increase in non-SMA (1.684 ± 0.197, *p* = 0.0366), SMA-carrier (2.350 ± 0.48, *p* = 0.0402), SMA1 (1.967 ± 0.25, *p* = 0.0010) and SMA2 (1.90 ± 0.264, *p* = 0.0176) conditions, compared to their corresponding untreated controls (Fig. 2A). No differences of SMN levels were observed between non-treated and CTRL-ASO cultures. Our results indicated that adding *SMN*-ASO to the culture medium at the onset of the differentiation cycle from neurospheres to MNs significantly increased SMN protein in control and SMA conditions. To investigate whether the SMN protein increase in *SMN*-ASO-treated MNs was associated with increased level of *SMN* transcripts, we quantified by RT-qPCR the *SMN* mRNA level in 20 days differentiated SMA1 and SMA2 MNs. *SMN* mRNA was increased in *SMN*-ASO treated SMA conditions (SMA1 1.738 ± 0.1521, *p* = 0.0007, SMA2 1.91 ± 0.21, *p* = 0.0012), compared to their respective non-treated control cells (Fig. 2B). This outcome indicated that *SMN*-ASO elevates *SMN* transcripts in SMA hiPSC-derived MNs.

Next, we explored whether *SMN*-ASO treatment increased Smn level in cultured SMA mouse MNs. To this end, WT and SMNΔ7 E13.5 spinal cords were obtained and submitted to MN isolation and culture protocol. After plating, 120 nM *SMN*-ASO or 120 nM CTRL-ASO were added to the culture medium. Six days after treatment, protein extracts were obtained and submitted to western blot using an anti-SMN antibody. Results showed that *SMN*-ASO increased Smn level in SMNΔ7 (2.64 ± 0.42) cells, compared to non-treated (*p* = 0.0025) or CTRL-ASO-treated MNs (*p* = 0.0041) (Fig. 2C). Taken together, results obtained from both experimental models indicated that *SMN*-ASO increased SMN level in SMA MNs. Since CTRL-ASO had no effect on SMN levels, we used only *SMN*-ASO to specifically increase SMN expression in the subsequent experiments.

Immunofluorescence protocol using an anti-SMN antibody was employed to analyze SMN levels in the soma and neurites of *SMN*-ASO-treated MNs. As expected, measuring relative fluorescence level revealed that SMN was increased in soma and neurites of *SMN*-ASO-treated SMA1 (soma: 42.74 ± 1.409, *p* < 0.0001; neurites: 3.68 ± 0.25, *p* = 0.02) and SMA2 (soma: 31.64 ± 1.78, *p* = 0.0008; neurites: 3.1 ± 0.18, *p* = 0.0017) cells, compared to their respective non-treated controls (SMA1 soma: 34.57 ± 1.024; neurites: 2.91 ± 0.22; SMA2 soma: 21.5 ± 1.25; neurites: 2.33 ± 0.15) (Fig. 2D). This result indicated that *SMN*-ASO elevated SMN in both soma and neurites MN compartments.

To study the effect of *SMN*-ASO treatment on the degenerative process of SMN-reduced MNs and its ability to prevent SMA phenotype, we next explored neurite degeneration in differentiated MNs. Neurite degeneration is associated with a variety of neuropathological conditions and correlates with impaired neuronal function [40–43]. Previous analysis of neurite swelling and blebbing showed that SMA differentiated MNs exhibited a higher index of neurite degeneration compared to the non-affected controls [16]. To evaluate the effect of ASO treatment, we quantified degenerated neurites using the ANDI macro for FIJI [40]. Immunofluorescence staining using an anti-β-III-Tubulin antibody was achieved in non-SMA, SMA-carrier, SMA1, and SMA2 20-day differentiated MNs. Results indicated that SMA (SMA1, 0.47 ± 0.025, *p* < 0.0001, *p* < 0.0001; SMA2, 0.42 ± 0.038, *p* = 0.0088, *p* = 0.0015) condition exhibited a significant increase in neurite degeneration (ND) index, compared to non-SMA (0.27 ± 0.015) and SMA-carrier (0.271 ± 0.022) conditions (Supplementary Fig. 4). No significant differences were observed between non-SMA and SMA-carrier (*p* > 0.9999). Notably, administration of 120 nM *SMN*-ASO reduced the ND index in treated cells (SMA1, 0.35 ± 0.023, *p* < 0.0001; SMA2, 0.199 ± 0.01, *p* < 0.0001), compared to the non-treated SMA condition (SMA1, 0.509 ± 0.026; SMA2, 0.31 ± 0.024) (Fig. 2E). These findings indicated that *SMN*-ASO treatment significantly diminishes neurite degeneration in SMA differentiated MNs.

### Effect of ASO treatment on apoptosis and autophagy markers altered in SMA differentiated MNs

**Fig. 3:**
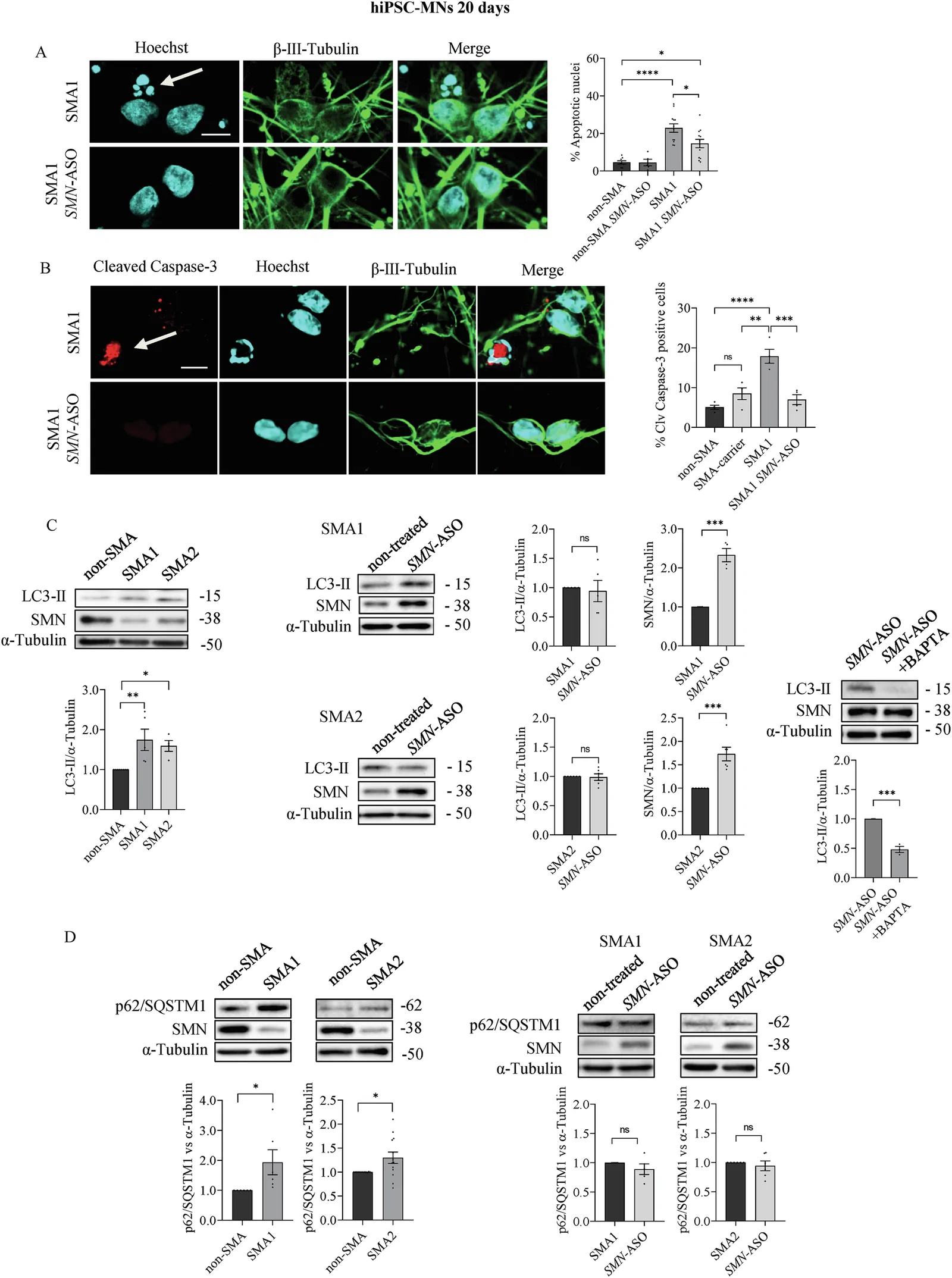
Effect of ASOs treatment on apoptosis and autophagy markers in SMA differentiated MNs. Representative immunofluorescence confocal images of 20-day differentiated non-treated or 120 nM *SMN*-ASO-treated SMA1 human MNs using an anti-β-III-Tubulin antibody (green) (**A**) or anti-Cleaved Caspase-3 antibody (red) and anti-β-III-Tubulin antibody (green) (**B**). Hoechst staining (cyan) was used to identify cell nucleus. Graph values represent the percentage of nuclei with apoptotic features (**A**) and the percentage of Cleaved Caspase-3 positive cells (**B**), corresponding to the quantification of at least 30 cells per condition from 3 independent hiPSCs differentiation cycles ± SEM. Arrows indicate apoptotic nucleus (**A**) and Cleaved Caspase-3 positive cells (**B**). Scale bar, 15 μm. **C**, **D** LC3-II and p62/SQSTM1 proteins were analyzed in 20-day differentiated non-SMA and non-treated or 120 nM *SMN*-ASO-treated SMA1 and SMA2 MNs. LC3-II was assessed in *SMN*-ASO SMA2 cells in the absence or presence of 10 µM BAPTA (**C**, right). Protein extracts were submitted to western blot using anti-LC3, anti-p62/SQSTM1, and anti-SMN antibodies. Membranes were reprobed using anti-α-tubulin antibody. Graph values represent LC3-II (**C**), SMN (**C**), and p62/SQSTM1 (**D**) versus α-tubulin relative to the non-SMA (**C**, **D**), non-treated SMA (**C**, **D**) or *SMN*-ASO SMA2 (**C**) conditions (normalized to 1) and correspond to the quantification of 4 independent hiPSCs differentiation cycles ± SEM. Asterisks indicate significant differences using one-way Anova with Tukey’s multiple comparisons post-test or Student *t* test (*p < 0.05, **p < 0.01, ***p < 0.001, ****p < 0.0001, ns *p* > 0.05).

To further study the effect of *SMN*-ASO treatment in intracellular mechanisms altered in SMA MNs, we next analyzed cellular events related to apoptosis and autophagy. Alterations in apoptotic pathway have been described in SMA MNs [16, 29]. Here we monitored the number of apoptotic nuclei in MNs differentiated from hiPSCs. Twenty-day differentiated non-SMA and SMA1 MNs were treated with 120 nM *SMN*-ASO and submitted to immunofluorescence protocol using an anti-β-III-Tubulin antibody. Hoechst staining was used to assess the percentage of nuclei presenting apoptotic features (chromatin condensation, nuclear fragmentation or blebbing). Fluorescence images were acquired using a 20x objective with the Olympus BX51 epifluorescence microscope, and measures were performed blinded and manually. SMA1 cultures showed a higher percentage of apoptotic nuclei (22.9 ± 2.2, *p* < 0.0001), compared to non-SMA (4.6 ± 0.87), which was significantly attenuated by ASO treatment (14.65 ± 2.2, *p* = 0.02) (Fig. 3A). We next analyzed the cleaved caspase-3 positive cells. SMA1 cultures showed a significant increase in the percentage of cleaved caspase-3 positive cells (17.89 ± 1.74), compared to the non-SMA (5.06 ± 0.536, *p* < 0.0001) and SMA-carrier (8.46 ± 1.47, *p* = 0.0015) controls. No differences were detected between non-SMA and SMA-carrier cells. Addition of *SMN*-ASO clearly reduced the percentage of cleaved caspase-3 positive cells in SMA1 (6.97 ± 1.24, *p* = 0.0004) compared to the untreated condition (Fig. 3B). These results together indicated that *SMN*-ASO treatment reduces apoptotic parameters in 20-day differentiated SMA MNs.

Previous findings reported autophagy alterations in several SMA models [15, 31, 32, 44], with increased L3C-II and p62/SQSTM1 protein levels in both mouse and human SMA MNs [15, 31, 45]. Therefore, we decided to analyze the ability of ASO treatment to regulate LC3-II and p62/SQSTM1 elevation. Control and SMA hiPSCs were differentiated to MNs and cell lysates were collected at 20-day post-differentiation and submitted to western blot using anti-LC3 and anti-p62/SQSTM1 antibodies. SMA MNs exhibited increased LC3-II (SMA1, 1.74 ± 0.266, *p* = 0.0050; SMA2 1.593 ± 0.13, *p* = 0.034) (Fig. 3C) and p62/SQSTM1 (SMA1, 1.94 ± 0.42, *p* = 0.0495; SMA2 1.3 ± 0.12, *p* = 0.0181) (Fig. 3D) compared to non-SMA condition. Notably, elevated levels of LC3-II and p62/SQSTM1 were not reduced in SMA conditions when 120 nM *SMN*-ASO was added to the culture medium. As shown in Fig. 3C, D, no differences in LC3-II (SMA1, 0.94 ± 0.18, *p* = 0.757; SMA2 0.988 ± 0.056, *p* = 0.84) and p62/SQSTM1 (SMA1, 0.89 ± 0.09, *p* = 0.2583; SMA2 0.94 ± 0.08, *p* = 0.504) were detected between treated and untreated SMA cells. ASO treatment effectively increased SMN protein level in SMA cells (SMA1, 2.328 ± 0.17, *p* = 0.0002; SMA2 1.73 ± 0.356, *p* = 0.0005), compared to the non-treated condition (Fig. 3C). These results indicated that *SMN*-ASO did not reduce LC3-II and p62/SQSTM1 protein level in 20-day differentiated SMA MNs.

To explore whether LC3-II level was modulated by intracellular calcium reduction in SMA MNs [46], *SMN*-ASO-SMA2 cells were incubated in the presence of the calcium chelator BAPTA. Ten  µM BAPTA-AM (Cat. No. B6769, Invitrogen) was added to the culture medium of ASO-treated SMA2 MNs, and western blot analysis was performed using the anti-LC3 antibody. Results in Fig. 3C (right) showed that LC3-II level was reduced in BAPTA-treated cells (0.48 ± 0.052, *p* = 0.0006) compared to the control condition. This result indicated that intracellular calcium reduction decreased LC3-II in ASO-treated SMA human differentiated MNs.

### *SMN*-ASO treatment does not prevent α-fodrin upregulation in differentiated human SMA MNs

**Fig. 4:**
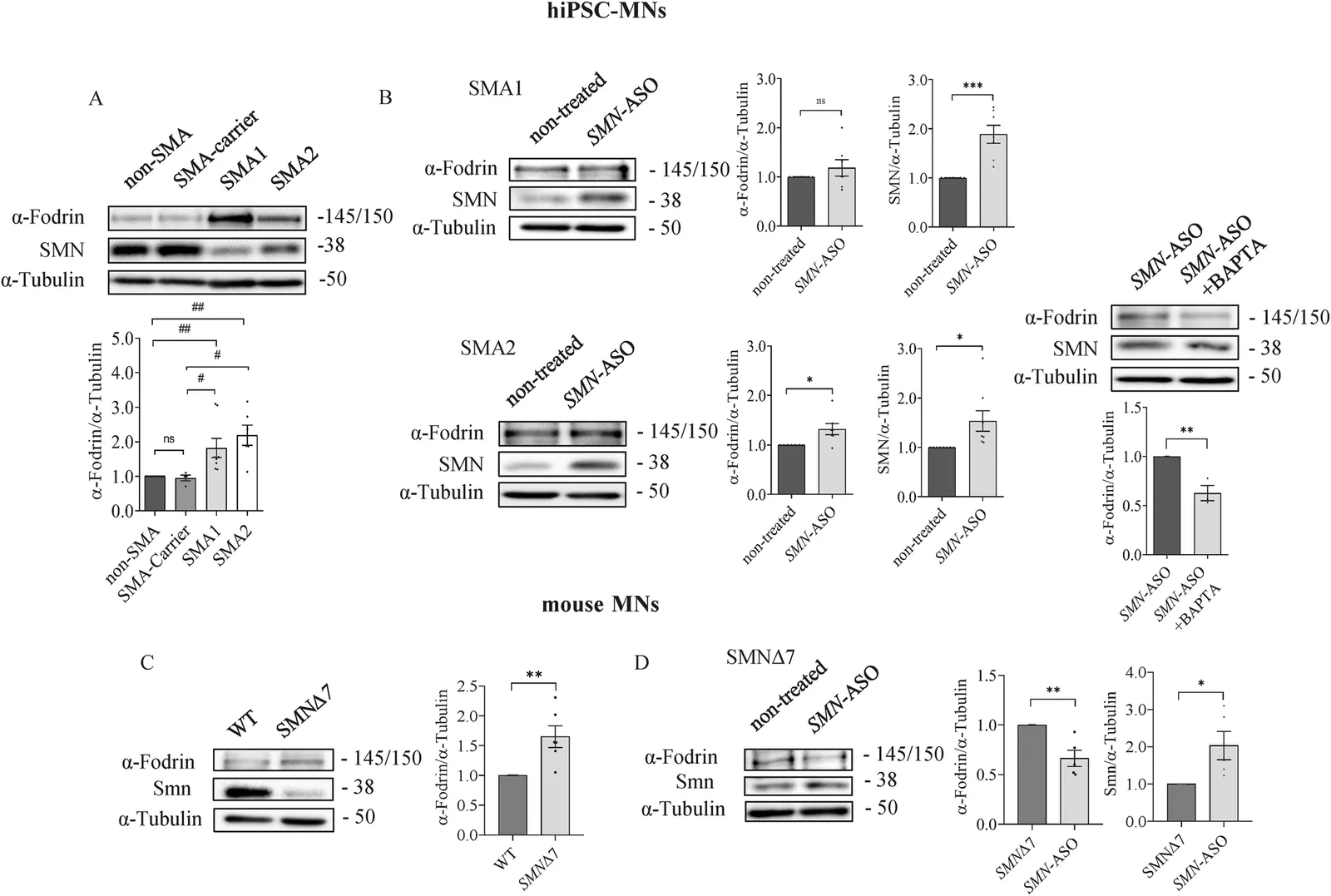
*SMN*-ASO treatment does not prevent α-fodrin upregulation in differentiated human SMA MNs. **A** Protein extracts of 20-day differentiated non-SMA, SMA-carrier, SMA1 and SMA2 MNs were collected. **B** SMA1 and SMA2 MNs were treated with 120 nM *SMN*-ASO or left untreated, 20 days after treatment cell lysates were obtained (left); ASO-treated SMA2 MNs were incubated or not with 10 µM BAPTA and protein extracts were collected (right). Western blot experiments were performed using anti-α-fodrin and anti-SMN antibodies (**A**, **B**). Membranes were reprobed using anti-α-tubulin antibody. Graph values represent the expression of α-fodrin 145/150 kDa and SMN versus α-tubulin relative to the non-SMA (**A**), non-treated SMA (**B**, left), and *SMN*-ASO SMA2 (**B**, right) conditions (normalized to 1) and correspond to the quantification of 7 (**A**, **B**, left) or 3 (**B**, right) independent hiPSCs differentiation cycles ± SEM. Total cell lysates of 6-day WT and SMN∆7 cultured mouse MNs (**C**) or non-treated or 120 nM *SMN*-ASO-treated SMN∆7 MNs (**D**) were analyzed by western blot using anti-α-fodrin and anti-SMN antibodies. Graph values represent the expression of α-fodrin 145/150 kDa and Smn versus α-tubulin relative to the WT (**C**) and non-treated SMN∆7 (**D**) conditions (normalized to 1) and correspond to the quantification of at least 4 independent experiments ± SEM. Symbols indicate differences using Kuskal-Wallis with Dunn’s multiple comparisons post-test (^#^*p* < 0.05, ^##^*p* < 0.01, ns *p* > 0.05) or Student *t* test (*p < 0.05, **p < 0.01, ***p < 0.001, ns *p* > 0.05).

Previous results showed that cultured SMA mouse and human differentiated MNs exhibit increased activity of the calcium-dependent protease calpain [36, 37]. Degradation of α-fodrin to the 145/150kDa-specific breakdown product is a well-characterized method to evaluate calpain activity by western blot [47]. Using an anti-α-fodrin antibody, we assessed the level of 145/150 kDa fragments in SMA MNs. Analysis of protein extracts obtained from 20-day differentiated MNs showed that 145/150 kDa α-fodrin fragments were significantly increased in SMA condition (SMA1, 1.82 ± 0.28, *p* = 0.0031 and *p* = 0.0431; SMA2, 2.19 ± 0.29, *p* = 0.001 and *p* = 0.0158), compared to the non-SMA and SMA-carrier conditions, respectively (Fig. 4A). No significant differences were observed between SMA-carrier (0.95 ± 0.08, *p* > 0.9999) versus non-SMA and between SMA1 versus SMA2 (*p* > 0.9999) in α-fodrin fragments. Unexpectedly, 120 nM *SMN*-ASO treatment did not prevent (SMA1, 1.19 ± 0.17, *p* = 0.28) or increase (SMA2, 1.32 ± 0.11, *p* = 0.0135) α-fodrin cleavage in SMA cultures, compared to non-treated SMA conditions (Fig. 4B). SMN protein was increased in ASO-treated SMA1 (1.889 ± 0.183, *p* = 0.0004) and SMA2 (1.54 ± 0.2, *p* = 0.0218), compared to the non-treated respective condition. These results indicated that in human SMA differentiated MNs, ASO treatment does not prevent α-fodrin degradation, suggesting increased calpain activity in these cells. To elucidate whether the increase of α-fodrin fragments was modulated by intracellular calcium reduction, we examined the effect of BAPTA-AM addition. *SMN*-ASO-treated SMA2 MNs were incubated or not with 10 µM BAPTA, and α-fodrin fragments were analyzed by western blot. Results indicated that BAPTA significantly reduced 145/150 kDa α-fodrin fragments in *SMN*-ASO SMA2 cells (0.63 ± 0.076, *p* = 0.0083) compared to the non-treated condition (Fig. 4B). Thus, reducing intracellular calcium diminished α-fodrin fragments accumulation in differentiated SMA human MNs treated with ASO.

Next, 145/150 kDa α-fodrin fragments were analyzed in mouse SMNΔ7 MNs. Isolated embryonic WT and SMNΔ7 MNs were cultured for 6 days and total cell lysates were collected. Western blot analysis of protein extracts revealed that α-fodrin fragments were increased in SMNΔ7 (1.65 ± 0.18, *p* = 0.0053), compared to WT control (Fig. 4C). To evaluate whether ASO treatment prevented 145/150 kDa α-fodrin increase, SMNΔ7 and 120 nM *SMN*-ASO-treated SMNΔ7 MNs were submitted to western blot analysis. Results showed that 145/150 kDa α-fodrin was significantly reduced in ASO-treated SMNΔ7 MNs (0.664 ± 0.082, *p* = 0.0085), compared to non-treated SMNΔ7 control (Fig. 4D). Treatment increased Smn protein level in SMNΔ7 MNs (2.034 ± 0.38, *p* = 0.00269). In contrast to the response observed in human SMA cells, these results indicated that ASO treatment in mouse SMNΔ7 MNs prevented the α-fodrin cleavage.

### *SMN*-ASO treatment increases Gemin3 protein in differentiated human SMA MNs

**Fig. 5:**
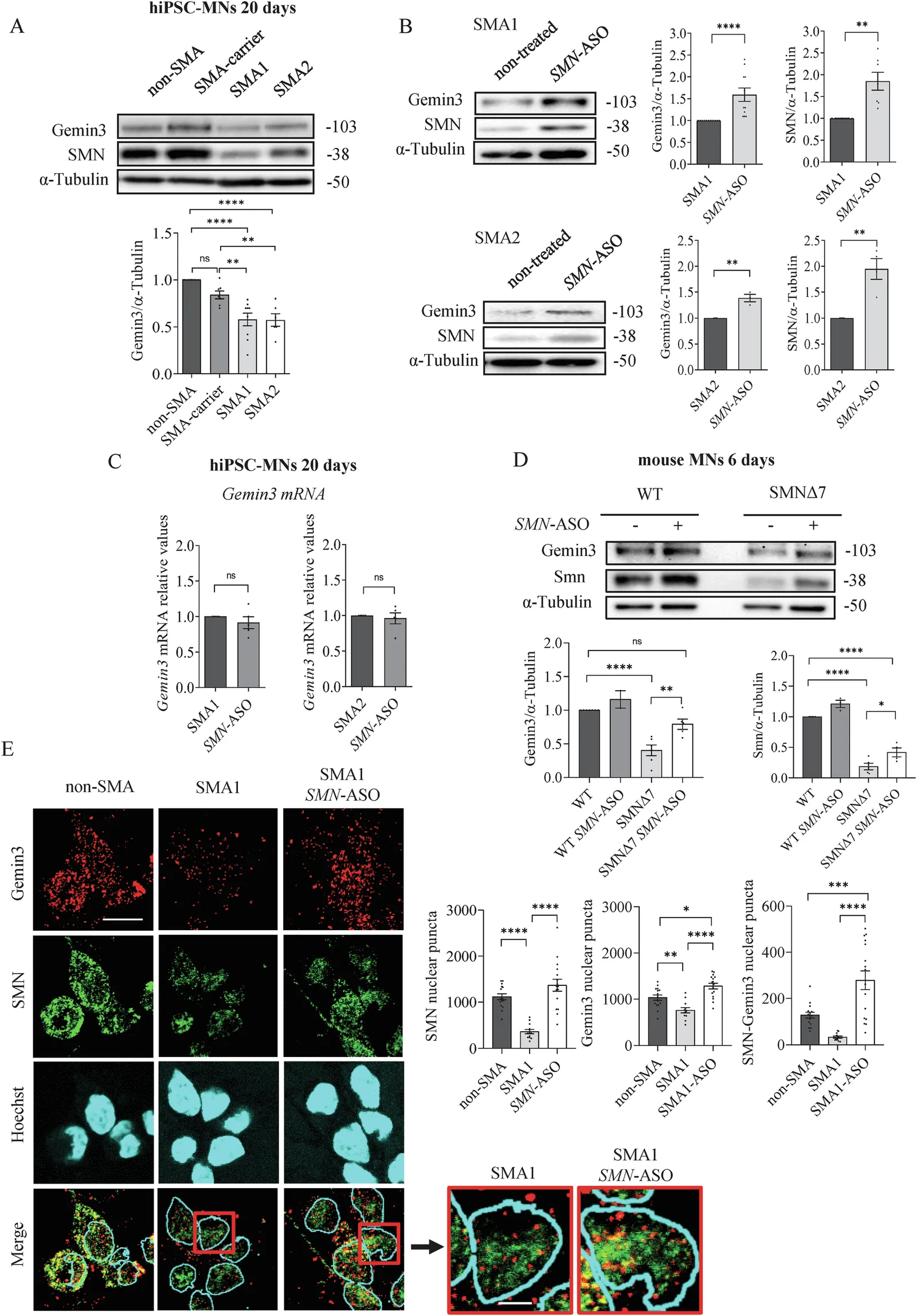
*SMN*-ASO treatment increases Gemin3 protein and mRNA level in differentiated human SMA MNs. Protein extracts of 20-day differentiated non-SMA, SMA-carrier, SMA1 and SMA2 MNs (**A**) or non-treated and 120 nM *SMN*-ASO-treated SMA1 and SMA2 MNs (**B**) were submitted to western blot using anti-α-Gemin3 and anti-SMN antibodies. Membranes were reprobed using anti-α-tubulin antibody. Graph values represent the expression of Gemin3 and SMN versus α-tubulin relative to the non-SMA (**A**) and non-treated SMA (**B**) conditions (normalized to 1) and correspond to the quantification of at least 4 independent hiPSCs differentiation cycles ± SEM. **C** Total mRNA from non-treated and treated SMA1 and SMA2 MNs was extracted, reverse transcribed to cDNA, and *Gemin3* was amplified through RT-qPCR. *GAPDH* gene was used as control. Graph values are the mean of *Gemin3* gene expression relative to the respective non-treated SMA condition (normalized to 1) from 5 independent hiPSCs differentiation cycles ± SEM. **D** Protein extracts of 6-day non-treated or 120 nM *SMN*-ASO-treated WT and SMN∆7 mouse MNs were obtained and submitted to western blot using anti-Gemin3 and anti-SMN antibodies. Graph values represent the expression of Gemin3 and Smn versus α-tubulin relative to the WT condition and correspond to the quantification of at least 4 independent experiments ± SEM. **E** Representative immunofluorescence confocal images of 20-day differentiated non-SMA and SMA human MNs using anti-Gemin3 (red) and anti-SMN (green) antibodies. Hoechst staining (cyan) was used to identify cell nucleus. Red squares show a magnification of SMA1 cells and cyan profile corresponds to the nucleus area where puncta were quantified. Graph values represent the number of SMN, Gemin3, or SMN-Gemin3 co-localized puncta per nucleus, corresponding to the quantification of 30-50 nuclei per condition from 3 independent hiPSCs differentiation cycles ± SEM. Scale bars, 15 μm and 5 μm. Asterisks indicate significant differences using one-way Anova with Tukey’s multiple comparisons post-test or Student *t* test (*p < 0.05, **p < 0.01, ***p < 0.001, ****p < 0.0001, ns *p* > 0.05).

Gemin3 protein is a core member of the SMN complex, regulates NF-κB survival pathway activation, and is reduced in MNs differentiated from human SMA hiPSCs [38]. In this context, we wanted to analyze whether ASO administration influenced Gemin3 level in SMA cells. Protein extracts of 20-day differentiated non-SMA, SMA-carrier, SMA1, and SMA2 MNs were obtained and submitted to western blot using an anti-Gemin3 antibody. As expected, Gemin3 protein level was significantly reduced in SMA1 (0.58 ± 0.07, *p* < 0.0001 and *p* = 0.0038) and SMA2 (0.57 ± 0.07, *p* < 0.0001 and *p* = 0.0076), compared to the non-SMA and SMA-carrier condition, respectively (Fig. 5A). No significant differences were observed in Gemin3 protein levels between SMA-carrier (0.84 ± 0.04, *p* = 0.9997) and non-SMA samples. Addition of 120 nM *SMN*-ASO to the culture medium significantly increased Gemin3 protein in SMA MNs (SMA1, 1.59 ± 0.15, *p* < 0.0001; SMA2, 1.38 ± 0.069, *p* = 0.0051), compared to non-treated SMA cells (Fig. 5B). SMN protein level was increased in both SMA1 (1.851 ± 0.205, *p* = 0.0014) and SMA2 (1.948 ± 0.201, *p* = 0.0033) conditions upon ASO treatment.

To explore whether the Gemin3 protein increase in SMA treated MNs was associated with Gemin3 transcript modification, we quantified *Gemin3* mRNA by RT-qPCR. *Gapdh* gene was used as a control. Results showed that ASO treatment did not increase *Gemin3* mRNA in SMA1 (0.914 ± 0.082, *p* = 0.33) and SMA2 (0.962 ± 0.077, *p* = 0.63) MNs, compared to the non-treated respective controls (Fig. 5C). These results together indicated that the observed Gemin3 protein increase after ASO treatment in SMA cells did not result from upregulation of *Gemin3* mRNA transcription.

To explore Gemin3 regulation in mouse SMA MNs, we analyzed the level of this protein in ASO-treated and non-treated WT and SMNΔ7 MNs. Protein extracts from 6-day cultured cells were submitted to western blot. As observed in human MNs, SMA mouse MNs exhibited reduced Gemin3 protein (0.403 ± 0.079, *p* < 0.0001), compared to the WT control (Fig. 5D). Additionally, when SMNΔ7 cells were treated with 120 nM *SMN*-ASO, Gemin3 protein was increased (0.792 ± 0.075, *p* = 0.0021), compared to the non-treated SMNΔ7. Treatment significantly elevated Smn protein level in SMNΔ7 *SMN*-ASO (0.417 ± 0.073, *p* = 0.026), compared to the SMNΔ7 (0.186 ± 0.053). These findings collectively indicated that Gemin3 protein increases after *SMN*-ASO treatment in both human and mouse SMA MNs.

Double-labeled immunofluorescence, using anti-Gemin3 and anti-SMN (rabbit anti-SMN/Gemin1 from Abcam, ab314895) antibodies on 20-day control and SMA1 differentiated MNs, detected Gemin3 and SMN puncta distributed throughout nucleus, cytoplasm, and neurite compartments. As expected, SMN and Gemin3 nuclear puncta were significantly reduced in SMA1 (SMN, 362.8 ± 47.39; Gemin3, 759.6 ± 56.13), compared to non-SMA cells (SMN, 1 116 ± 65, *p* < 0.0001; Gemin3, 1034 ± 57.78, *p* = 0.0084) (Fig. 5E). When SMA1 cells were treated with 120 nM *SMN*-ASO, the level of SMN and Gemin3 puncta was increased (SMN, 1 366 ± 130.7, *p* < 0.0001; Gemin3, 1 283 ± 61.31, *p* < 0.0001), compared to non-treated SMA1 condition. To ascertain whether SMN and Gemin3 were co-localized in the nucleus of these cells, the overlap of SMN and Gemin3 fluorescent puncta were examined (see Supplementary Fig. 5). As observed in Fig. 5E, the number of co-localized SMN-Gemin3 puncta in *SMN*-ASO-treated SMA1 condition (278.7 ± 40.35) was clearly augmented, compared to SMA1 (33.42 ± 4.736, *p* < 0.0001). Together, these results indicated that ASO treatment enhances SMN and Gemin3, as well as their expression, at the same locations into the nuclear compartment of SMA MNs.

### *SMN*-ASO treatment increases the levels of the NF-κB pathway members IKKβ and RelA in SMA MNs

**Fig. 6:**
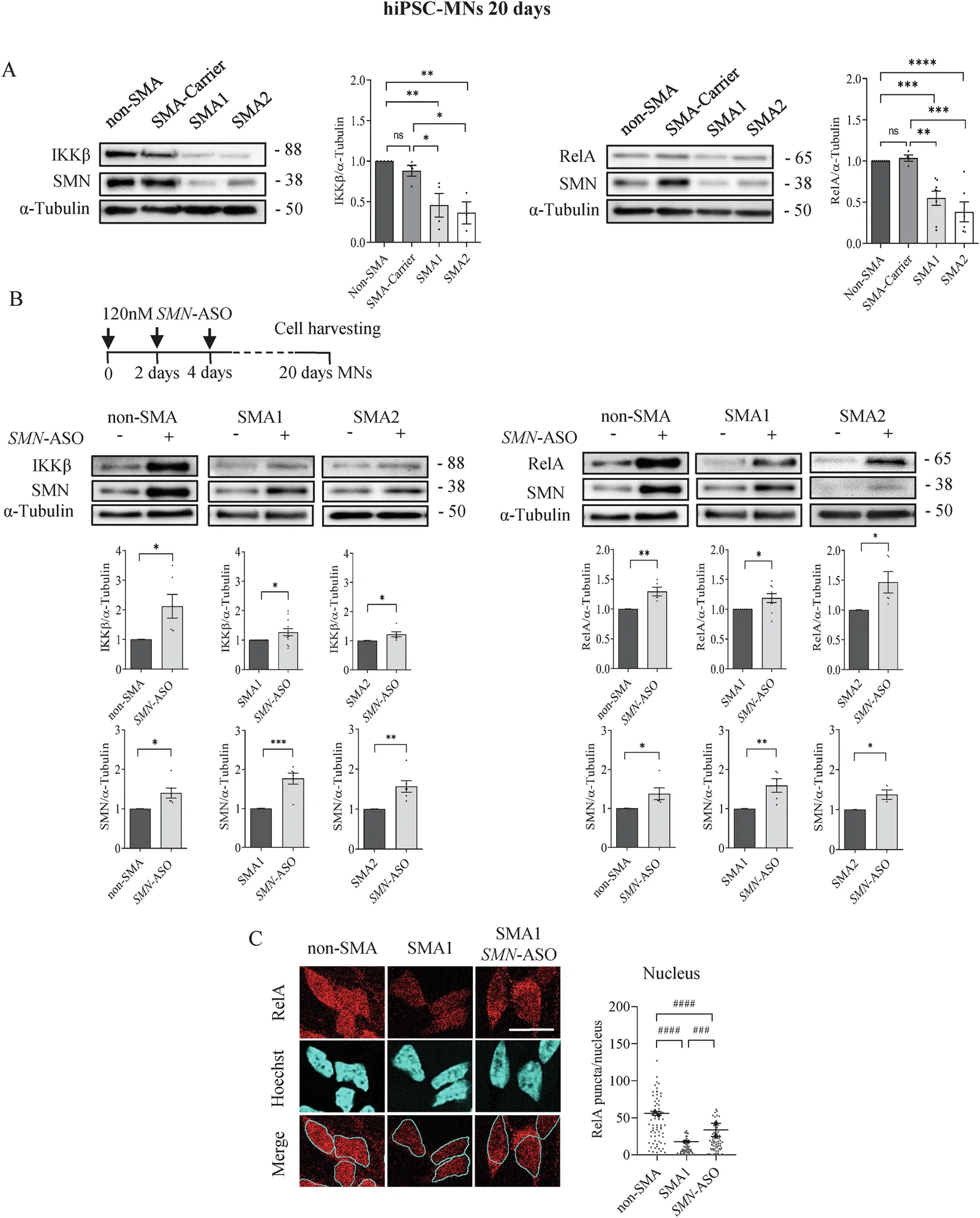
*SMN*-ASO treatment increases IKKβ and RelA protein level in SMA MNs. Protein extracts of 20-day differentiated non-SMA, SMA-carrier, SMA1 and SMA2 MNs (**A**) and non-treated and 120 nM *SMN*-ASO-treated SMA1 and SMA2 MNs (**B**) were submitted to western blot using anti-IKKβ, anti-RelA and anti-SMN antibodies. Membranes were reprobed using anti-α-tubulin antibody, used as a loading control. Graph values represent the expression of IKKβ, RelA and SMN versus α-tubulin relative to the non-SMA (**A**) and non-treated (**B**) conditions (normalized to 1) and correspond to the quantification of at least 4 independent hiPSCs differentiation cycles ± SEM. **C** Representative immunofluorescence confocal images of 20-day differentiated non-SMA and SMA1 MNs using anti-RelA (red) antibody. Hoechst staining (cyan) was used to identify cell nucleus. Cyan profile corresponds to the nucleus area where RelA puncta were quantified. Graph values represent the number of RelA puncta per nucleus, corresponding to the quantification of 60-75 nuclei per condition from 3 independent hiPSCs differentiation cycles ± SEM. Scale bar, 15 μm. Symbols indicate significant differences using one-way Anova with Tukey’s multiple comparisons post-test, Student *t* test (*p < 0.05, **p < 0.01, ***p < 0.001, ****p < 0.0001), or Kuskal-Wallis with Dunn’s multiple comparisons post-test (^###^*p* < 0.001, ^####^*p* < 0.0001).

Previous results indicate that NF- κB signaling pathway is associated to MN survival and SMN protein regulation [48]. SMN-deficient MNs show disrupted NF-κB signaling and Gemin3 knockdown regulates IKKβ and RelA protein level [34, 38]. The increase of Gemin3 observed after ASO treatment in SMA MNs (Fig. 5) led to the analysis of NF-κB regulation in this experimental condition. Total cell lysates from 20-day differentiated control and SMA MNs were submitted to western blot using anti-IKKβ and anti-RelA antibodies. As expected, IKKβ and RelA protein levels were reduced in SMA1 (IKKβ: 0.4575 ± 0.1447, *p* = 0.0045 *p* = 0.0317; RelA: 0.55 ± 0.08631, *p* = 0.0005, *p* = 0.0025) and SMA2 (IKKβ: 0.3633 ± 0.1362, *p* = 0.0026, *p* = 0.0151; RelA: 0.3833 ± 0.1209, *p* < 0.0001, *p* = 0.0002), compared to non-SMA and SMA-carrier (Fig. 6A). No differences in IKKβ and RelA level were observed between non-SMA and SMA-carrier conditions. Treatment of non-SMA and SMA cells with 120 nM *SMN*-ASO induced a significant increase in IKKβ (non-SMA, 2.118 ± 0.3995, *p* = 0.0188; SMA1, 1.269 ± 0.1218, *p* = 0.0378; SMA2, 1.217 ± 0.0841, *p* = 0.0276) and RelA (non-SMA, 1.294 ± 0.0715, *p* = 0.0034; SMA1, 1.188 ± 0.07501, *p* = 0.0235; SMA2, 1.466 ± 0.1826, *p* = 0.0339) level compared to the non-treated conditions (Fig. 6B). After NF-κB pathway stimulation, RelA is released, relocates to the nucleus, and binds to the κB DNA elements, resulting in gene induction or gene repression [49]. To evaluate nuclear RelA after *SMN*-ASO treatment in SMA1 cells, 20-day differentiated MNs were submitted to an immunofluorescence protocol using an anti-RelA antibody. Confocal images were obtained and RelA puncta were measured in the nucleus compartment. RelA was significantly reduced in SMA1 (8.74 ± 1.12) compared to non-SMA cells (45.65 ± 3.52, *p* < 0.0001). *SMN*-ASO addition significantly increased the number of RelA puncta in SMA1 cells (21.42 ± 2.02; *p* = 0.0001) compared to the non-treated condition (Fig. 6C). Despite the observed increase in nuclear RelA in the treated cells, RelA levels remained below those detected in the non-SMA group (*p* < 0.0001). These results together indicated that ASO treatment regulated NF-κB members in SMA MNs.

## Discussion

The development of targeted therapies to increase SMN protein represents a significant advance in SMA disease management. Nevertheless, therapeutic response differs between patients, leading to changes in disease phenotypes and increasing variability in SMA clinical presentation [1]. The intracellular mechanisms altered in SMN-reduced MNs, causing cell degeneration and death, are still under study. Understanding the final pathways leading to MN collapse is a central objective of SMA research. In the present work, we analyzed the effect of ASO treatment on altered intracellular pathways in SMA differentiated human MNs derived from iPSCs. Our final objective is to determine the ability of ASO to modify the intracellular landscape occurring in these cells in order to identify potential new targets for SMA combinatorial therapies. Even though recent evidence indicates that SMA is a multi-organic disease, MNs are extremely sensitive to SMN protein reduction, causing their degeneration in early stages of the disease [50]. In vitro models to study the disease can provide significant support to the multiple existing in vivo models, both non-murine and mouse [51]. Patient-derived cells can be used to gain valuable insights on the local mechanistic effects of reduced SMN expression. Here, we presented a robust protocol to differentiate human MNs derived from iPSCs, based on the differentiation protocol published by Du and colleagues [52]. Analysis of SMN protein levels in these cells revealed a clear reduction in SMA MNs compared to both non-SMA and SMA-carrier. We noticed that SMA-carrier condition exhibited significantly reduced levels of SMN at the initial period of MN differentiation compared to non-SMA, but at the later differentiation stages SMN level was similar to non-SMA. SMN reduction in heterozygotes during early differentiation may reflect haploinsufficiency of *SMN1* gene, compounded by inefficient *SMN2* splicing. Subsequent normalization to control levels likely arises from adaptive *SMN2* upregulation and/or SMN stabilization [53].

In our experiments, ASO treatment efficiently increased SMN protein in SMA differentiated MNs, which was maintained after 20 days of differentiation. Moreover, ASO addition prevented neurite degeneration, indicating that SMN increase is able to counteract this biological effect observed in SMN-reduced MNs. In our experimental paradigm, we examined some of the previously described altered intracellular pathways in SMA MNs. The analyzed mechanisms are involved in mediating MN survival or degeneration [36, 37, 48, 54]. Gemin3 is a multifunctional protein, a core member of the SMN complex, and regulates MN homeostasis [55, 56]. Previous evidence suggests the interdependence of Gemin3 and SMN [57]. For instance, Gemin3 knockdown reduces SMN protein in non-SMA MNs, and Gemin3 is decreased in SMN-reduced MNs [38]. Here, we showed that ASO treatment increased Gemin3 protein and the Gemin3-SMN co-localization in the nucleus of 20-day SMA differentiated MNs. Nevertheless, ASO treatment did not increase *Gemin3* mRNA in these cells. These findings suggest that SMN increase after ASO treatment may stabilize Gemin3 protein through their interaction. Notably, previous studies have provided evidence that Gemin2 and Gemin3 are stabilized by SMN in neurites and growth cones SMN-Gemin complexes [12, 58]. In Hela cells, Gemin3 interacts directly with SMN and the spliceosomal Sm proteins in vitro [56]. In SMA, SMN interactions and the formation of complexes are abolished, compromising the correct function of the splicing machinery [10]. Moreover, the recruitment of Gemin3 and SMN into the multi-protein complex increases their resistance to proteasomal degradation, which denotes that their stability is higher when they are part of the SMN complex, compared to the unassociated protein pools [59]. After ASO treatment, increased Gemin3 and Gemin3-SMN interaction in SMA MNs may contribute to the stabilization of these proteins and the SMN-complex, which in turn may regulate the biogenesis, assembling, metabolism of the snRNP, pre-mRNA splicing, mRNA stabilization, trafficking and transcription. Alternatively, Gemin3 protein is also involved in roles that do not directly involve RNA [55]. It is known that Gemin3 reduction contributes to the increase of apoptotic cell death through p53 activation [60]. In SMA differentiated MNs, ASO treatment reduced the percentage of apoptotic nuclei and caspase-3 positive cells, suggesting that Gemin3 increase may contribute to apoptosis regulation in these cells. Moreover, we observed an increase of IKKβ and RelA protein members of the NF-κB pathway, a key cellular signaling mechanism that plays crucial roles in neurons, regulating synaptic plasticity, neurite outgrowth, and neuroprotection [61–63]. NF-κB inhibits apoptosis, promotes cell survival, and increases SMN protein expression in MNs [48]. Reducing the level of Gemin3 negatively regulates the NF-κB pathway and increases neurite degeneration in cultured MNs [38]. Post-treatment restoration of the pathway may play a critical role in promoting cellular recovery and mitigating neurite collapse.

Autophagy is a highly conserved process involved in the intracellular homeostasis maintenance [64]. SMN-depleted MNs display autophagy alterations including increased autophagosome number and reduced autophagy flux [15, 31, 32, 45]. Our findings showed that LC3-II and p62/SQSTM1 protein increase detected in SMA MNs was not prevented by ASO treatment. Although alterations in these proteins have been linked to defects in the autophagy pathway, our study did not directly assess whether the autophagy machinery remains functionally compromised. This limitation could be addressed in future studies by including direct flux measurements and autophagy/lysosomal markers co-localization experiments.

Calcium homeostasis alterations have been associated with autophagy dysregulation [65]. Evidence from our previous and current studies show a reduction in LC3-II protein after addition of the calcium chelator BAPTA-AM in SMA cultured MNs [46], indicating a link between intracellular calcium level and autophagy. Elevated intracellular calcium levels activate the protease calpain, which cleaves several targets, including SMN and autophagy-related proteins [35, 36, 45, 66]. Present results indicated that 145/150 kDa α-fodrin fragments did not decrease in human differentiated SMA MNs after ASO treatment. However, intracellular calcium chelation reduced α-fodrin cleavage in these treated cells, suggesting increased Calpain-1 activity. By contrast, *SMN*-ASO treatment reduced the α-fodrin fragments in cultured mouse SMNΔ7 MNs. Human iPSC-derived and mouse primary spinal cord MN models display differences in the expression of genes related to voltage-gated calcium channels, and electrophysiology studies have shown large differences in spontaneous firing activity [67]. In addition, divergences in calcium regulation have been described between mouse and chick cultured MNs [68]. These observations highlight the intrinsic differences between the in vitro mouse and human MNs and the significance of employing multiple models to elucidate the molecular mechanisms underlying MN degeneration in SMA.

**Fig. 7:**
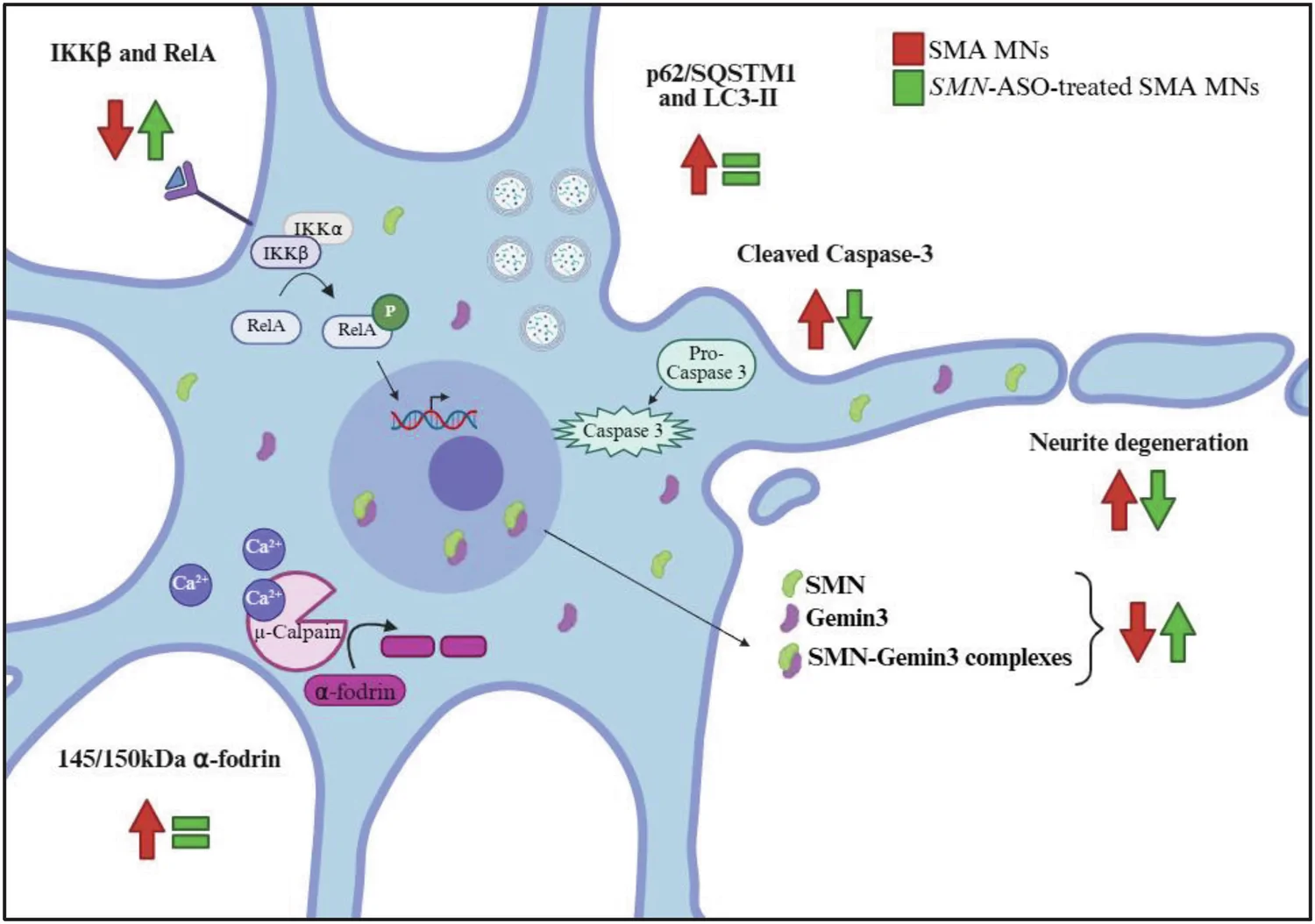
Schematic representation of the effect of *SMN*-ASO treatment on altered intracellular pathways in differentiated human SMA MNs. Red arrows indicate increased or reduced proteins of intracellular pathways and neurite degeneration in non-treated SMA differentiated human MNs from hiPSCs. Green symbols indicate the effect of ASO treatment on the level of these proteins and on the percentage of collapsed neurites. After ASOs treatment, the percentage of neurite degeneration and Cleaved caspase-3 protein levels decreased; SMN, Gemin3, and IKKβ/RelA protein levels increased; the increased levels of α-Fodrin 145/150 kDa and LC3-II were unchanged (Created in BioRender. Pazos, M (2026) https://BioRender.com/6c3hy9b).

In our study, *SMN*-ASO treatment prevented neurite degeneration, reduced apoptosis markers, and increased SMN, Gemin3, IKKβ, and RelA protein level in 20‑day differentiated SMA MNs. Nevertheless, the autophagy markers (LC3-II and p62/SQSTM1) and the products of calpain activation (145/150 kDa α-fodrin) were altered (Fig. 7). The present results suggest incomplete normalization of intracellular pathways at the time point examined. Future experiments, including in vivo models and analysis across earlier and later stages, would clarify whether these changes are transient, delayed‑to‑resolve, or truly persistent, and would help address some of the limitations of our study. The residual intracellular alterations underscore the importance of distinguishing between phenotypic rescue and complete molecular normalization, thereby framing our findings in a more balanced clinical context. Combinatorial therapeutic strategies targeting residual intracellular changes may have translational relevance, as they could help identify novel therapeutic approaches in the future. Disease-modifying therapies have substantially slowed the natural progression of the disease and improved SMA patients’ quality of life. Considering that SMA is a complex disease, investigating the molecular events occurring after treatment may contribute to understand the mechanisms underlying emerging disease variants.

## Materials and methods

### SMA animals

The SMA mice model FVB⋅Cg-*Grm 7* ^Tg(SMN2)89Ahmb^ Smn 1^TM1Msd^ Tg(SMN2*delta7)4299Ahmb/J (IMSR_JAX:005025) (The Jackson Laboratory, Bar Harbor, Maine, USA) was used for the study. SMNΔ7 (Smn-/-; SMN2+/+; SMN∆7+/+) mutants were obtained by crossing heterozygous (Smn+/-; SMN2+/+; SMN∆7+/+) animals. Littermates SMNΔ7 and control (wild type, WT) (Smn+/+; SMN2+/+; SMNΔ7+/+) were used for the experiments.

For primary MN cultures, E13 embryos were genotyped. The Phire Tissue Direct PCR Master Mix (Thermo Fisher Scientific, Waltham, MA, USA) was used for genomic DNA extraction and PCR setup, using the following primers: WT forward 5′-CTCCGGGATATTGGGATTG-3′; SMA reverse 5′-GGTAACGCCAGGGTTTTCC-3′; and WT reverse 5′-TTTCTTCTGGCTGTGCCTTT-3′.

All procedures were approved by the Universitat de Lleida Advisory Committee on Animal Services (CEEA02-02/23) and by the Generalitat de Catalunya, Departament d’acció climàtica, alimentació i agenda rural (CEA-OH/12150/2, project number 12150). Experiments were performed in accordance with the Spanish Council on Animal Care guidelines.

### Mouse spinal cord MN isolation and culture

Primary cultures of mouse MNs were achieved using spinal cords of genotyped WT and SMNΔ7 E13 embryos as previously described [68–70]. Isolated MNs were plated in laminin-coated four-well tissue culture dishes (Nunc, Thermo Fisher Scientific) for western blot analysis (80,000 cells/well). Cells were maintained in neurobasal medium (Gibco, Thermo Fisher Scientific) supplemented with B27 (2% v/v) (Gibco), horse serum (2% v/v) (Gibco), L-glutamine (0.5 mM) (Gibco), 2-mercaptoethanol (25 µM) (Sigma, Saint Louis, MO, USA), and a cocktail of neurotrophic factors: BDNF (1 ng/ml), GDNF (10 ng/ml), CNTF (10 ng/ml), and HGF (10 ng/ml) (Peprotech, Gibco, Thermo Fisher Scientific). Twenty-four h after plating, 2 µg/ml of aphidicolin (Sigma) was added to the culture medium and maintained during the experiment.

### Differentiation of human-induced pluripotent stem cells to MNs

Four human induced pluripotent stem cell (hiPSC) lines were used: (a) the GM23411 (RRID:CVCL_T816, Coriell Institute for Medical Research, Camden, NJ, USA) hiPSCs obtained from a healthy 3-month-old individual, as control (non-SMA condition); (b) the GM24474 (RRID:CVCL_T826, Coriell) hiPSCs obtained from the GM03814 (RRID:CVCL_F173, Coriell Institute) fibroblast cell line of a clinically unaffected mother of two affected children (5 *SMN2* copies; heterozygous for deletion exon7-8 in *SMN1*), as SMA-carrier condition; (c) the GM23240 (RRID:CVCL_C878, Coriell Institute) hiPSCs obtained from the GM03813 (Coriell Institute, RRID:CVCL_F172) fibroblast cell line of a 3-year-old SMA type 2 patient (3 *SMN2* copies; deletion exon7-8 in *SMN1*), as SMA2 condition (patient was initially classified as SMA type 1, but several data points supported re-classification to SMA type 2); and (d) the CS84iSMA-nxx (Cedar Sinai, Los Angeles, USA, RRID:CVCL_A9T6) hiPSCs obtained from the GM10684 (Coriell Institute, RRID:CVCL_W564) lymphoblast cell line of a 6-month-old SMA type 1 patient (2 *SMN2* copies; deletion exon7-8 in *SMN1*), as SMA1 condition (Supplementary Fig. 1).

Human iPSCs were differentiated to MNs as previously described [37, 52]. Cell lines were expanded on Geltrex (Sigma)-coated plates in Essential 8 medium (Gibco) and neuroepithelial cells and MN progenitors were generated, following the published protocol. To induce MN differentiation, MN progenitors were detached with Accutase (Gibco) and cultured in suspension in MN induction medium, DMEM/F12: NBM plus 1:1 supplemented with B27+; L-glutamine (all from Gibco); 0.1 mM ascorbic acid (Sigma); 3 µM CHIR99021; 2 µM SB431512; 2 µM DMH1 (all from Cayman, Ann Harbor, MI, USA); 0.5 µM retinoic acid (Sigma); and 0.1 µM purmorphamine (Cayman). After 6 days, neurospheres were dissociated with Accumax (Invitrogen, Waltham, MA, USA) and seeded in laminin-coated plates in MN maturation medium: MN induction medium supplemented with 0.1 µM Compound E (Sigma), 20 ng/ml ciliary neurotrophic factor (CNTF), and 20 ng/ml Insulin-like growth factor 1 (IGF-1) (both from Peprotech, Gibco, Thermo Fisher Scientific). Dissociated neurospheres were plated in laminin-coated 1 cm^2^ glass coverslips placed into four-well dishes for immunofluorescence experiments (25,000 cells/well), in laminin-coated four-well dishes for western blot analysis (90,000 cells/well), or in laminin-coated six-well dishes (Falcon, Corning Incorporated, Corning, NY, USA) for RT-qPCR (500,000 cells/well) (Supplementary Fig. 1).

### Antisense oligonucleotide (ASO) design and treatment

Antisense oligonucleotides (ASOs) were synthesized by Integrated DNA Technologies-BVBA (Leuven, Belgium) with the same sequence and modification as Nusinersen, *SMN*-ASO (5′-TCACTTTCATAATGCTGG-3′) and CTRL-ASO (5′-TTAGTTTAATCACGCTCG-3). Briefly, all nucleotides were modified at every base with phosphorothioate backbones and 2′-O-methoxyethyl group, and cytosines with an additional 5-methyl modification. Lyophilized ASOs were reconstituted in water at 100 mM and stored at −20 °C. For in vitro treatment, 120 nM of *SMN*-ASO or CTRL-ASO were added to the culture medium 2 or 3 h after plating. Cells were re-treated on days 2 and 4 at the same concentration (see Supplementary Fig. 3).

### Western blot analysis

Western blots were performed as previously described [37, 68]. Cell lysates were resolved in sodium dodecyl-sulfate polyacrylamide (SDS-PAGE) gels and transferred onto polyvinylidene difluoride (PVDF) Immobilon-P transfer membranes (Millipore, Burlington, MA, USA) using a Trans-Blot turbo transfer system (Bio-Rad, Hercules, CA, USA). Membranes were blocked and then incubated with the following primary antibodies at the indicated dilutions: anti-SMN (1:5000, Cat. No. 610646, RRID:AB_397973) from BD Transduction Laboratories, Franklin Lakes, NJ, USA; anti-α-fodrin (1:4000, Cat. No. FG6090, RRID:AB_2050678) from Enzo Life Science, Farmingdale, NY, USA; anti-LC3 (1:1000, Cat. No. 2775, RRID:AB_915950) and anti-p62/SQSTM1 (1:1000, Cat. No. 5114, RRID:AB_10624872), both from Cell Signaling Technology, Danvers, MA, USA; anti-Gemin3 (12H12) (1:1000, Cat. No. sc-57007, RRID:AB_629518) for human samples and anti-Gemin3 (D-5) (1:500, Cat. No. sc-271853, RRID:AB_10708130) for mouse samples, both from Santa Cruz Biotechnology Dallas, TX, USA; and anti-IKKβ (1:1000, Cat. No. 8943, RRID:AB_11024092) and anti-RelA (1:1000, Cat. No. 6956, RRID:AB_10828935), both from Cell Signaling Technology. To control the specific protein content per lane, membranes were reprobed with anti-α-tubulin antibody (1:50,000, Cat. No. T5168, RRID:AB_477579, Sigma). Blots were developed using Luminata^TM^ Forte Western HRP Substrate (Millipore) and the ChemiDoc^TM^ Imaging System (Bio-Rad).

### RNA isolation and quantitative RT-PCR

For RT-qPCR experiments, SMA1 and SMA2 hiPSCs were differentiated to MNs. Cells were plated in laminin-coated six-well culture dishes at a density of 500,000 cells/well (Falcon, Corning Incorporated, Corning, NY, USA). Total RNA was extracted using the RNeasy Mini Kit (Quiagen, Hilden, Germany) following the manufacturer’s guidelines. The assays were performed in a CFX96 Real-Time System (Bio-Rad) using the iTaq^TM^

Universal SYBR Green One-Step Kit (Bio-Rad). Eighty nanograms of total RNA were used for each individual RT-qPCR reaction. Experiments were performed using the human Gemin3 specific primers: forward (5′-GCTGGCCGTTTTGGTACATTG-3′) and reverse (5′-TGCACAGCAGCTTTAACTTCC-3′). The specific primers for human glyceraldehyde-3-phosphate dehydrogenase (GAPDH): forward (5′-TGCACCACCAACTGCTTAG-3′) and reverse (5′- AGAGGCAGGGATGATGTTG-3′) were used as internal control. Samples were obtained from at least three independent hiPSCs differentiation cycles. Quantification was completed using Bio-Rad CFX Manager real-time detection system software (version 3.1, Bio-Rad). Relative fold change gene expression levels were calculated with the formula 2^-(ΔΔCq).

### Immunofluorescence

For immunofluorescence experiments, cultured cells were fixed with 4% paraformaldehyde (Sigma) for 10 min and post-fixed with cold methanol (Sigma) for 10 additional min. Cells were permeabilized using 0.3% Triton X-100 and incubated for 30 min in blocking solution (10% BSA and 0.3% Triton X-100 in PBS). The primary antibodies used for the experiments were anti-SMN (1:400, Cat. No. 610646, RRID:AB_397973) from BD Biosciences; anti-SMN/Gemin1 (1:50, Cat. No. ab314895, EPR25897-27) (Fig. 5E), anti-HB9 (1:75, Cat. No. ab92606, RRID:AB_10561593), and anti-ChAT (1:100, Cat. No. ab18736, RRID:AB_2244867) from Abcam, Cambridge, UK; and anti-β-III-Tubulin (1:400, Cat. No. 5568, RRID:AB_10694505), anti-RelA (1:200, Cat. No. 6956, RRID:AB_10828935), and anti-cleaved caspase-3 (1:100, Cat. No. 9661, RRID:AB_2341188) from Cell Signaling Technology. The primary antibody was diluted in blocking solution and incubated overnight at 4 °C. After washing, the secondary antibody was added. The secondary antibodies used were anti-mouse ALEXA555 (1:500, Cat. No. A21422, RRID:AB_2535844) and anti-rabbit ALEXA488 (1:500, Cat. No. A11008, RRID:AB_143165), both from Invitrogen (Waltham, MA, USA); and anti-rabbit ALEXA647 (1:500, Cat. No. 711-606-152, RRID:AB_2340625) and anti-sheep DyLight488 (1:500, Cat. No. 713-486-147, RRID:AB_2340741), both from Jackson ImmunoResearch (West Grove, PA, USA). Hoechst (1:400, Sigma) staining was used to identify nuclear localization. Samples were mounted using Mowiol (Calbiochem, San Diego, CA, USA) medium. Images were obtained with the Olympus FV1000 (Tokyo, Japan) or the Nikon Eclipse Ti2 AX with NSPARC (Amstelveen, The Netherlands) confocal microscope. Image acquisition was performed at 1024 × 1024-pixel resolution.

SMN fluorescence intensity analysis was performed blinded using the NIH ImageJ software [71]. β-III-Tubulin and Hoechst images were binarized and used to delimit the cell nucleus, soma, cytoplasm or neurite compartments. Defined areas were transferred to the corresponding SMN fluorescence images (see Supplementary Fig. 2). In the selected areas, the total sum of pixel values (Integrated Density, IntDen) was assessed. Three non-fluorescent surrounding regions were measured to set up the background readings. Fluorescence intensity was calculated using the following formula: Corrected Total Cell Fluorescence (CTCF) = IntDen – (ROI area × mean fluorescence of the background). SMN fluorescence intensity in neurites was quantified using CTCF values normalized to neurite area: CTCF/neurite area (µm^2^).

For SMN and Gemin3 puncta quantification, fluorescent images were acquired and cell nucleus compartment was defined using Hoechst binarized images. SMN or Gemin3 images were binarized and particles were measured using the Analyze particles tool in the nucleus compartment. To evaluate co-localization of SMN and Gemin3 particles in the nucleus, the logical AND operation of the Image calculator tool (ImageJ) was used (see Supplementary Fig. 5).

### Neurite degeneration measures in cultured MNs

Cells plated on laminin-coated coverslips were fixed and subjected to the immunofluorescence protocol using an anti-β-III-Tubulin antibody. Hoechst staining was used to identify nuclear localization. Fluorescence images were acquired using a 20× objective with the Olympus BX51 epifluorescence microscope under identical illumination and data capture conditions. For quantitative analysis, 10 images (1376 × 1104 pixels) of randomly sampled regions were recorded across 3 independent hiPSC differentiation cycles per condition. Neurite degeneration was evaluated using the open-source ANDI Macro for FIJI [40]. Hoechst staining and β-III-Tubulin immunolabeling from the same images were imported and subjected to binarization. Binarized nuclei were digitally withdrawn from the β-III-Tubulin binary images, thereby isolating an image containing only neuritic structures (Supplementary Fig. 4). Particles with an area between 0 and 4 pixels were excluded to minimize background. The area corresponding to fragmented neurites (observed in red) was determined using the FIJI Analyze Particles algorithm to measure particles with a circularity between 0.2 and 1.0 and size between 5 and 10,0000 pixels. The total area occupied by neurites was then quantified by applying the Measure tool to estimate the total black area. The neurite degeneration (ND) index was calculated as the ratio of the fragmented neurite area to the total neurite area. ND index values ranged from 0 to 1.

### Statistical analysis

The experiments were performed at least three independent times: three separate mouse offspring for primary MN cultures or three separate hiPSC differentiation cycles. Values were expressed as mean ± estimated standard error of the mean (SEM). Statistical analysis was achieved using GraphPad Prism, version 9.4.1 (GraphPad Software Inc, San Diego, CA, USA). Normality of the data was tested with Shapiro-Wilk test. When data distribution was normal, differences between two groups were assessed by two-tailed Student *t* test and differences between more than two groups by one-way ANOVA with Tukey’s multiple comparisons test. When data did not follow a normal distribution, non-parametric two-tailed Mann-Whitney test was used to compare differences between two groups and non-parametric Kruskal-Wallis with Dunn’s multiple comparisons test for more than two groups. Values were considered significant when *p* < 0.05. In graphs, statistical significance is shown with asterisks (*) when parametric tests were applied or with hash symbol (#) when non-parametric tests were applied.

## Supplementary information

Supplementary Figures

Uncroped original western blot

## Data Availability

All data generated or analyzed during this study are included in this published article and its supplementary information.
