# Supplementary material for: Antisense oligonucleotides treatment uncovers differences in the modulation of dysregulated intracellular pathways in Spinal Muscular Atrophy motoneurons
Source: Cell Death Dis. 2026 Jul 13;17(1):869. doi: 10.1038/s41419-026-09105-1 (PMC13642960; doi:10.1038/s41419-026-09105-1)
Supplement: Supplementary file 1 [file 41419_2026_9105_MOESM1_ESM.pdf]

Supplementary Figure 1

Condition	Cell line	Parent cell line and cell type	Age	Genetics	Patient classification
**Non-SMA**	GM23411*B	Fibroblast (GM00041)	3 months old	Not reported	Healthy individual
**SMA-carrier**	GM24474	Fibroblast (GM03814)	Not reported	*SMN1*: heterozygous for exon 7-8 deletion *SMN2*: 5 copies	Clinically unaffected
**SMA1**	CS84iSMA-nxx	Lymphoblastoid cell line (GM10684)	6 months old	*SMN1*: homozygous of exon 7-8 deletion *SMN2*: 2 copies	SMA type 1 patient
**SMA2**	GM23240*B	Fibroblast (GM03813)	3 years old	*SMN1*: homozygous of exon 7-8 deletion *SMN2*: 3 copies	SMA type 2 patient (re-classified) * Initially classified as SMA type 1

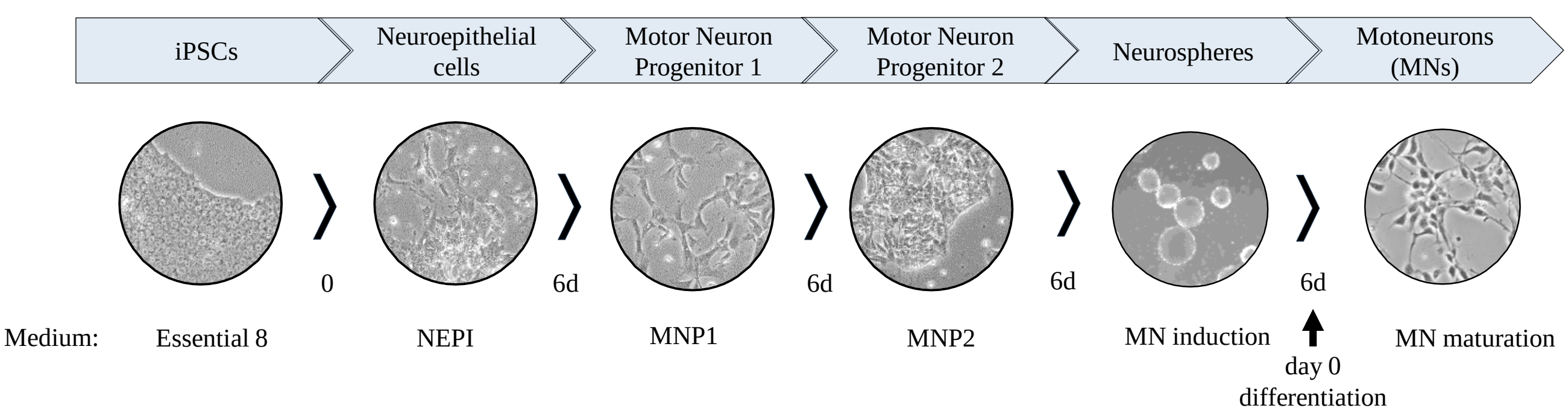

Suplementary Figure 1. Differentiation of human-induced pluripotent stem cell lines (hiPSCs) to motoneurons (MNs). Table summarizing patient iPSCs including parent cell line, age, genetics and patient classification. Schematic timeline of the differentiation protocol. Images illustrate each stage of differentiation with the specific medium used for neural induction and MN differentiation. Cells were maintained six days in each specific medium. The step of neurospheres disaggregation and plating was considered MN differentiation day 0.

Supplementary Figure 2

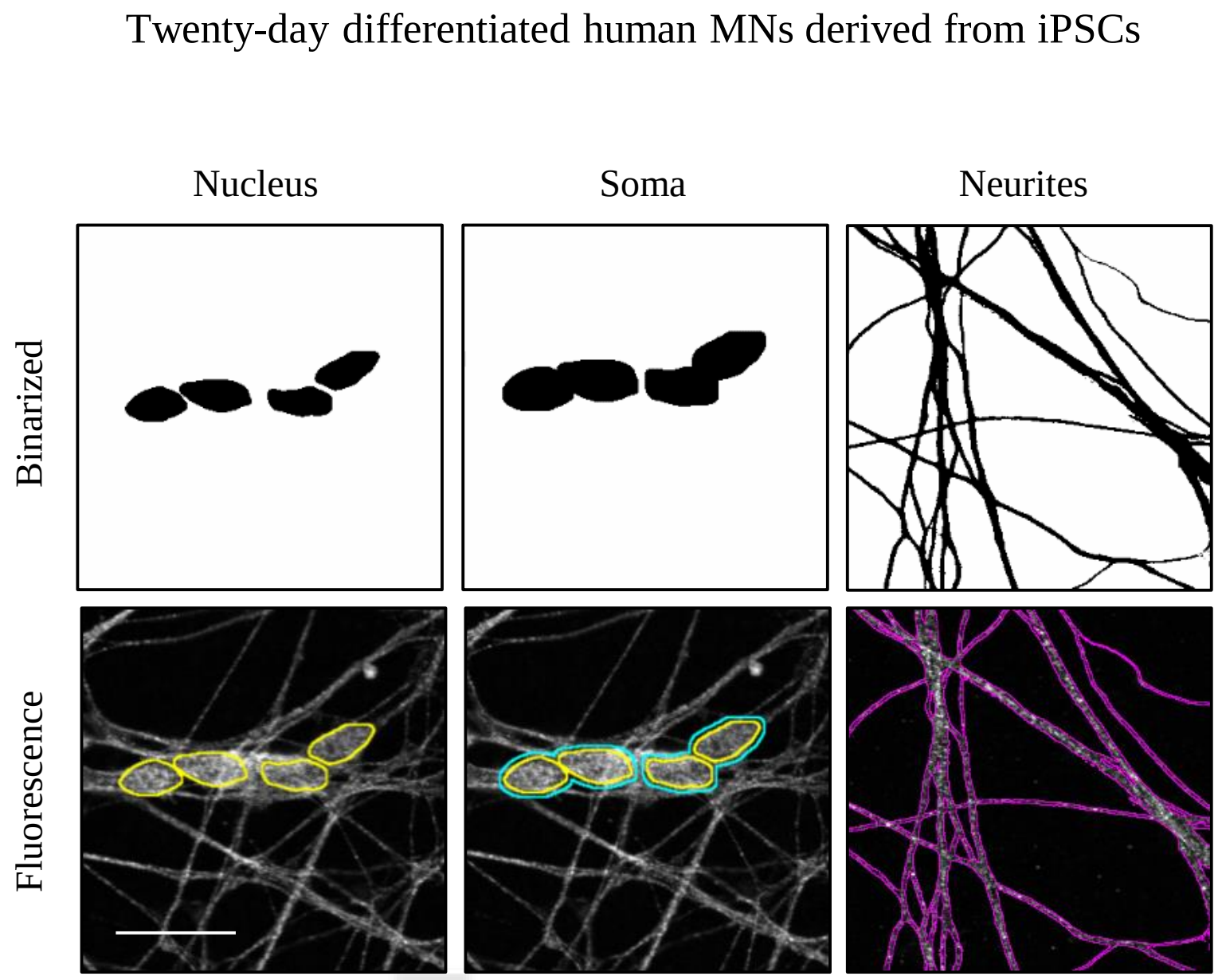

Suplementary Figure 2. Representative images of 20-day differentiated human MNs: binarized images of nuclei, somas and neurites (top) corresponding to the original fluorescent images (bottom). MNs were immunolabeled for SMN and β-III-Tubulin and counterstained with Hoechst nucleic acid dye. Images were subjected to automated binarization, and "Selections" of nuclei, somas or neurites were created using Hoechst or β-III-Tubulin fluorescent labeling. To determine the mean of SMN fluorescence in the specific cell compartments, these "Selections" were restored in original images, and the mean of grey values (Fluorescence) corresponding to SMN staining was measured.

Supplementary Figure 3

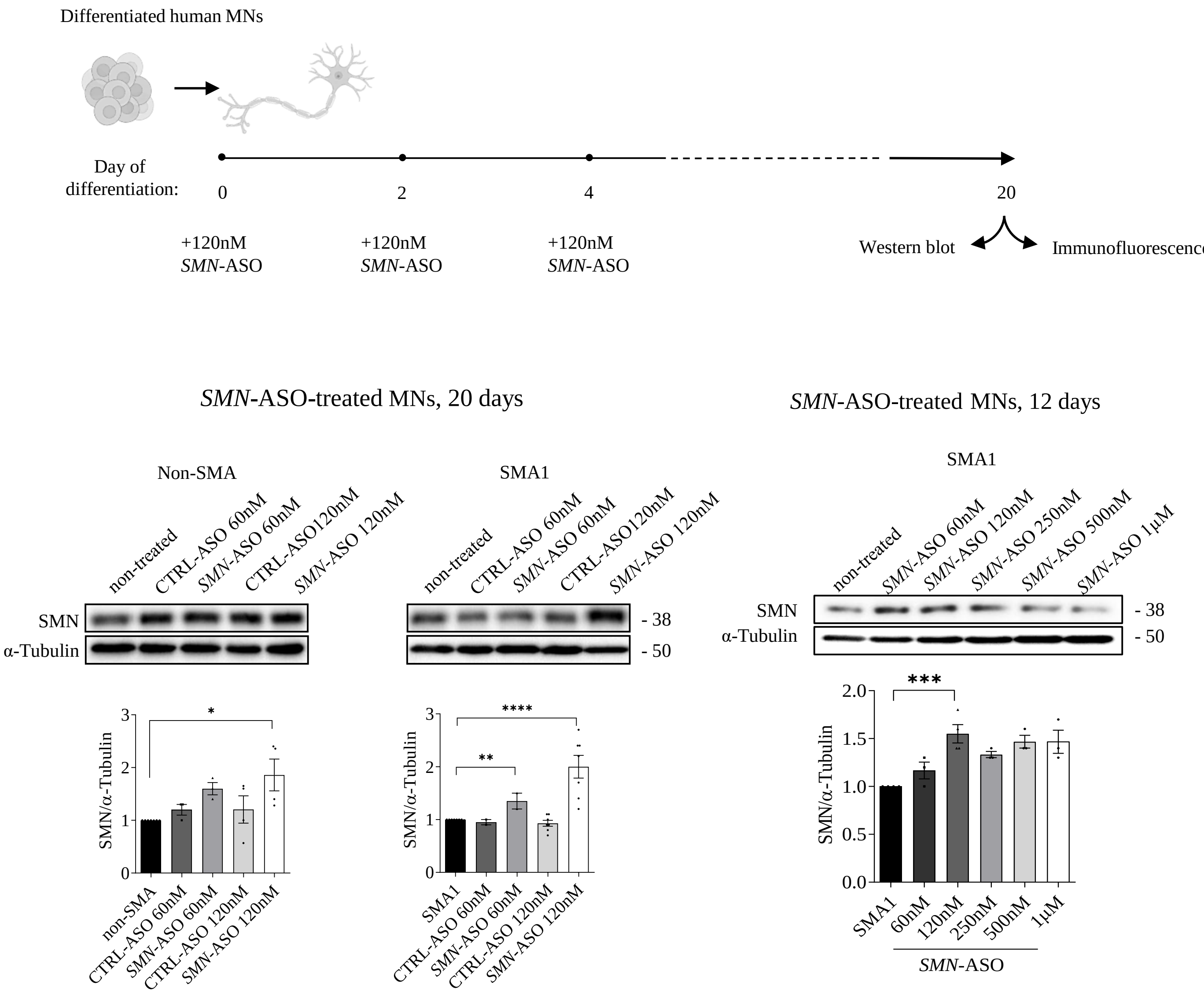

Suplementary Figure 3. Timeline of *SMN*-ASO treatment in human differentiated MNs. Dose-response analysis to identify the optimal ASO concentration. Non-SMA and SMA1 MNs were treated with 60nM or 120nM of *SMN*-ASO or CTRL-ASO. After 20 days, protein extracts were submitted to western blot using an anti-SMN antibody. Results indicated that 120nM *SMN*-ASO produced the greatest increase in SMN protein (left). To explore the effect of higher doses, SMA1 cells were treated with 60nM, 120nM, 250nM, 500nM or 1μM of *SMN*-ASO. The maximum effective dose was 120nM (right). Graph values represent the expression of SMN versus α-tubulin ± SEM. Asterisks indicate significant differences using one-way Anova with Tukey multiple comparisons post-test (**p*<0.05, ***p*<0.01, ****p*<0.001, *****p*<0.0001).

Supplementary Figure 4

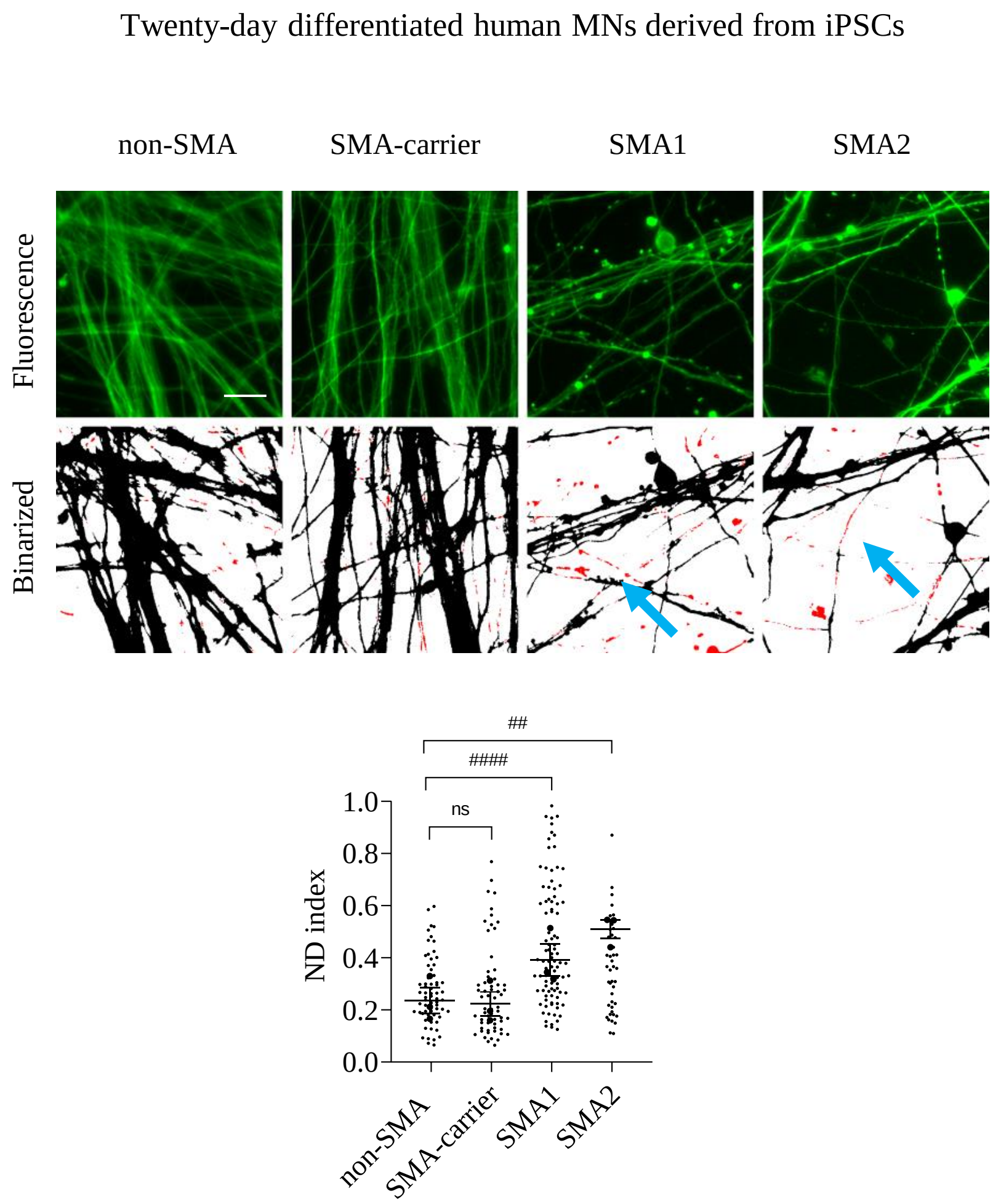

Supplementary Figure 4. SMA differentiated human MNs displayed increased neurite degeneration. Representative images of 20-day differentiated SMA and non-SMA MNs submitted to immunofluorescence protocol using an anti- β-III-Tubulin antibody. Hoechst staining was used to identify cell nucleus. Using the ANDI macro, β-III-Tubulin and Hoechst images were binarized and somas were removed. Total neurites and fragmented neurite areas were measured and Neurite Degeneration (ND) index was calculated. Scale bar, 30µm. Symbols indicate significant differences using the non-parametric Kruskal-Wallis with Dunn's multiple comparisons test ($^{\#\#}p<0.01$, $^{\#\#\#\#}p<0.0001$).

Supplementary Figure 5

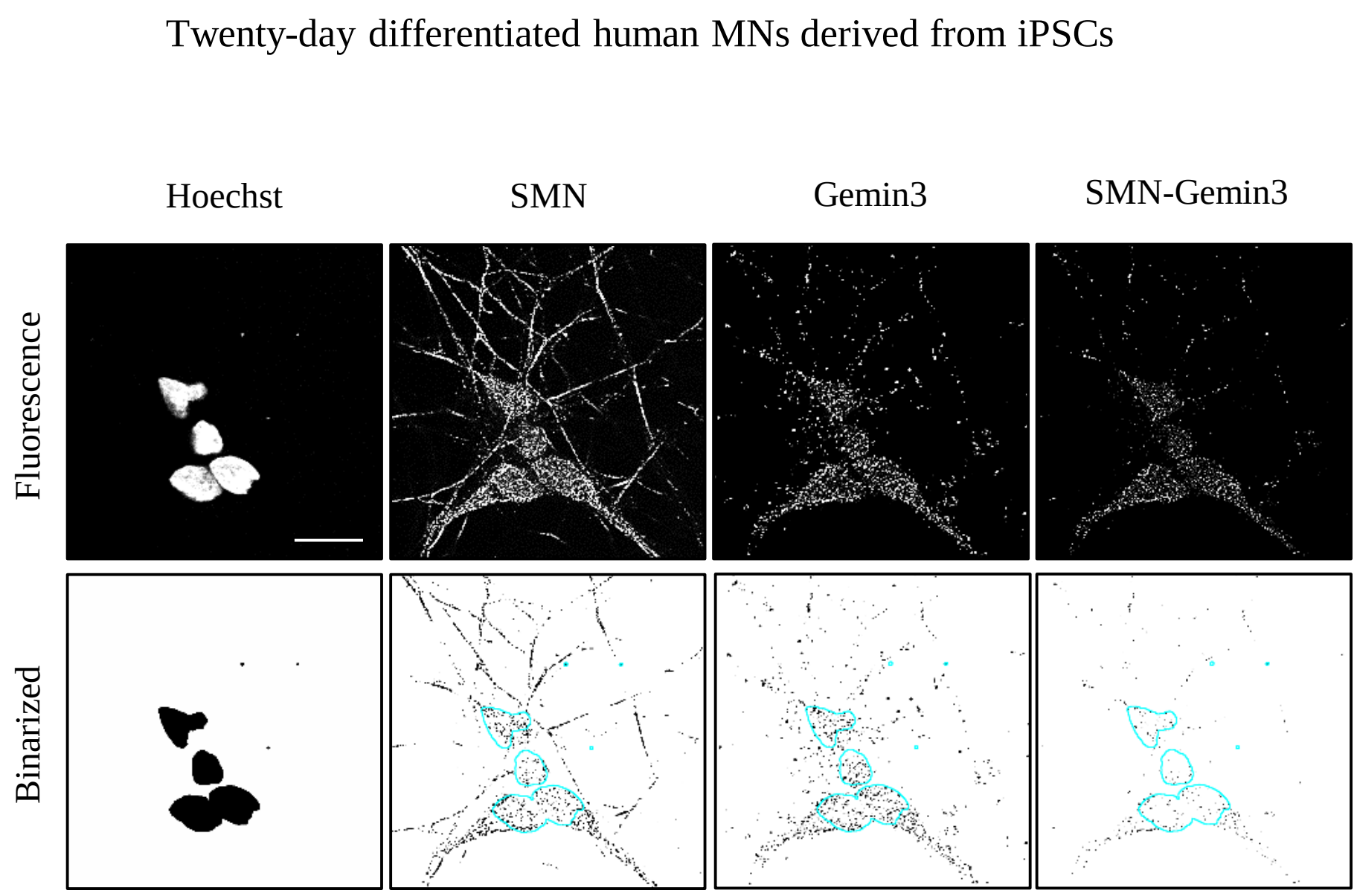

Supplementary Figure 5. Representative images of 20-day differentiated human MNs. Immunofluorescence protocol was performed using anti-SMN, anti-Gemin3 antibodies and Hoechst staining. Binarized images of Hoechst, SMN, Gemin3 and SMN-Gemin3 co-localized puncta were generated. "Image Calculator" tool was used to create binary images corresponding to the SMN-Gemin3 co-localized puncta. Hoechst binarized images were used to create "Selections" corresponding to the nuclear compartment. Puncta were measured in the selected nuclear area.
