# Supplementary material for: Antisense oligonucleotides treatment uncovers differences in the modulation of dysregulated intracellular pathways in Spinal Muscular Atrophy motoneurons
Source: Cell Death Dis. 2026 Jul 13;17(1):869. doi: 10.1038/s41419-026-09105-1 (PMC13642960; doi:10.1038/s41419-026-09105-1)

Full and uncropped western blots for Fig 1b, non-SMA Lane 1, SMA-carrier Lane 2, SMA1 Lane 3, and SMA2 Lane 4 are on the figures

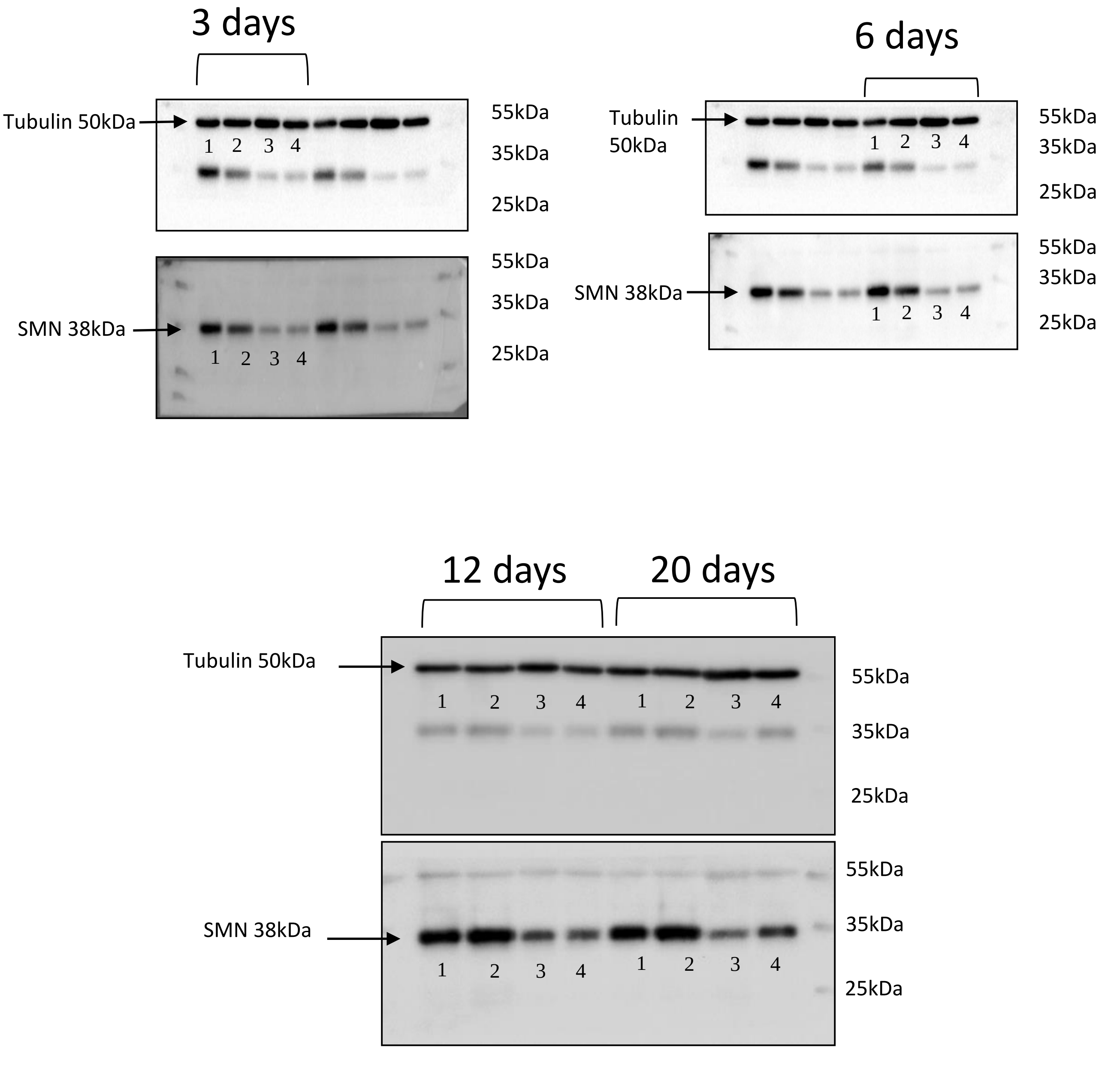

Full and uncropped western blots for Fig 2a, non-treated Lane 1, CTRL-ASO Lane 2, and *SMN*-ASO Lane 3 are on the figures

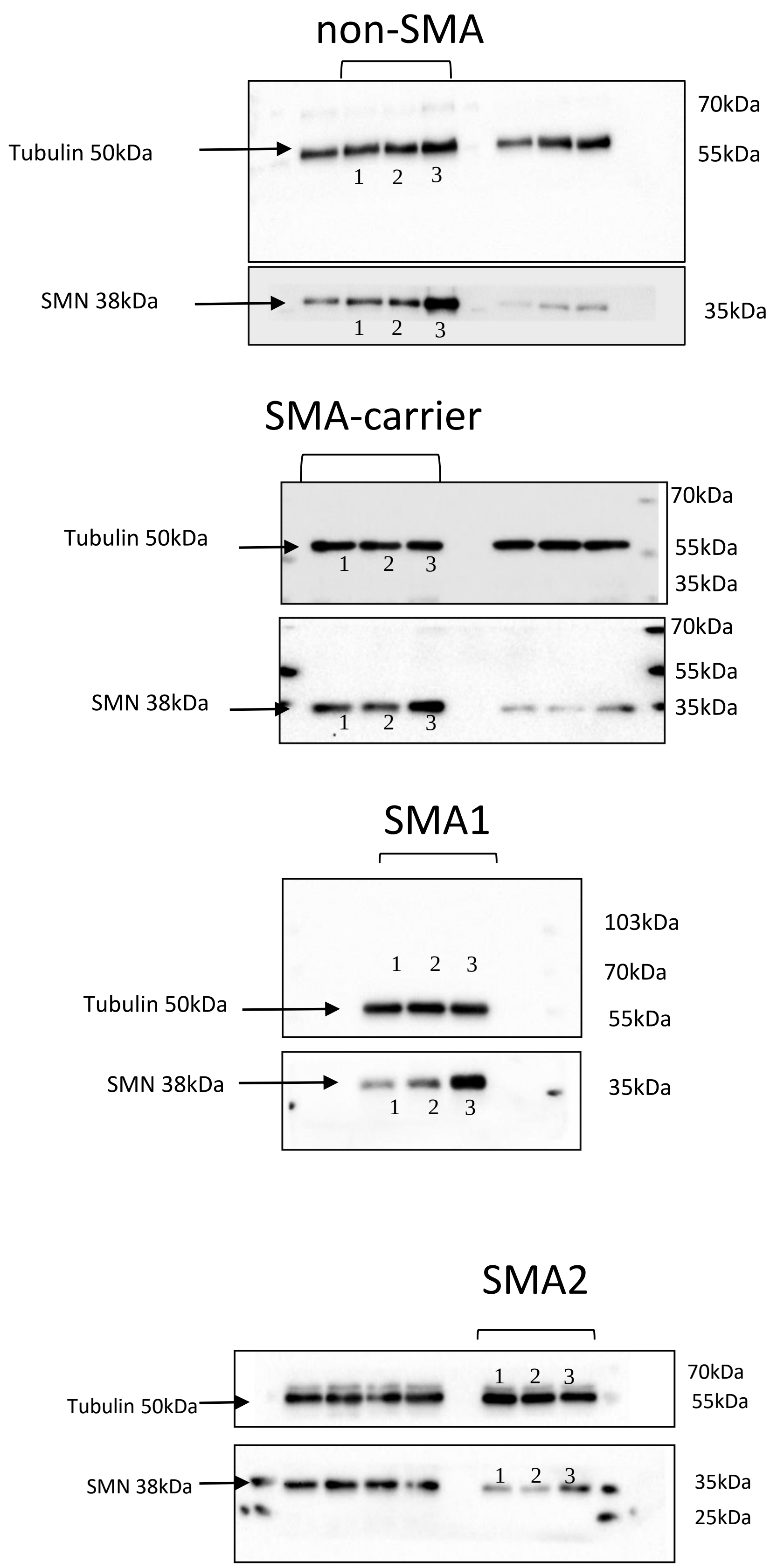

Full and uncropped western blot for Fig 2c, non-treated WT Lane 1, non-treated SMNΔ7 Lane 2, CTRL-ASO SMNΔ7 Lane 3, and *SMN*-ASO SMNΔ7 Lane 4 are on the figure

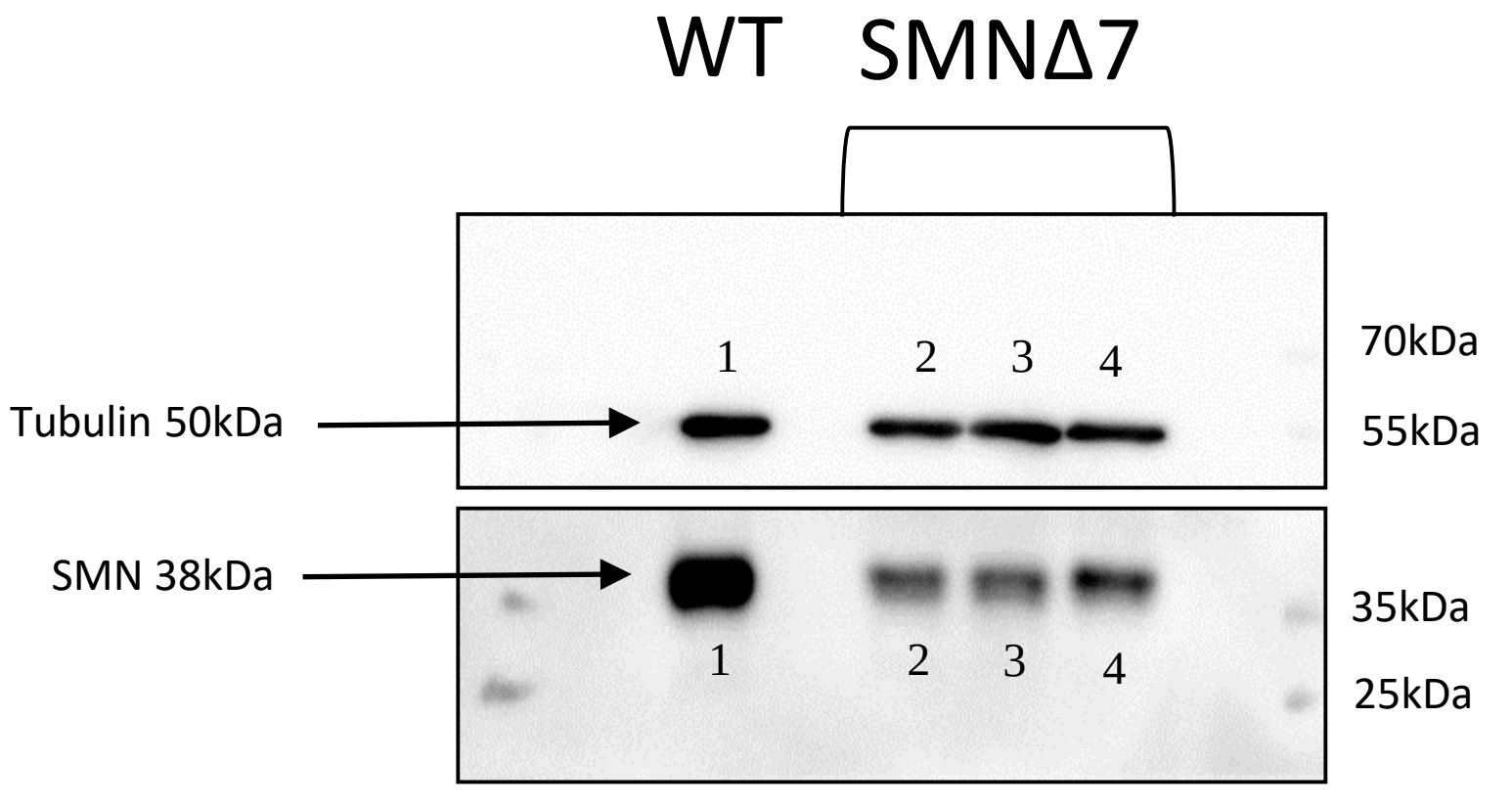

Full and uncropped western blots for Fig 3c, non-SMA Lane 1, SMA1 Lane 2, SMA2 Lane 3, SMA1 non-treated Lane 2.1, SMA1 *SMN*-ASO Lane 2.2, SMA2 non-treated Lane 3.1, SMA2 *SMN*-ASO Lane 3.2, and SMA2 *SMN*-ASO+BAPTA Lane 3.3 are on the figures

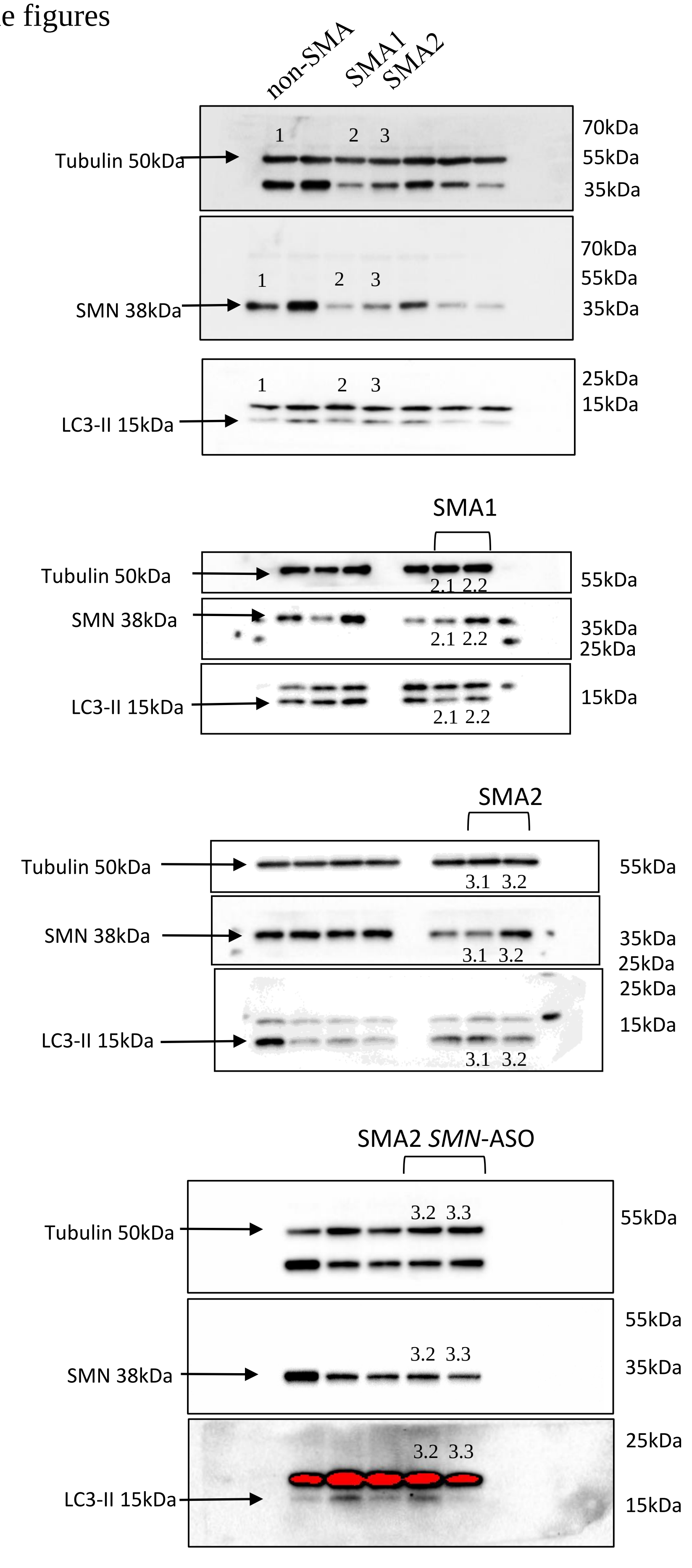

Full and uncropped western blots for Fig 3d, non-SMA Lane 1, SMA1 Lane 2, non-SMA Lane 3, SMA2 Lane 4, SMA1 non-treated Lane 2.1, SMA1 *SMN*-ASO Lane 2.2, SMA2 non-treated Lane 3.1, and SMA2 *SMN*-ASO Lane 3.2 are on the figures

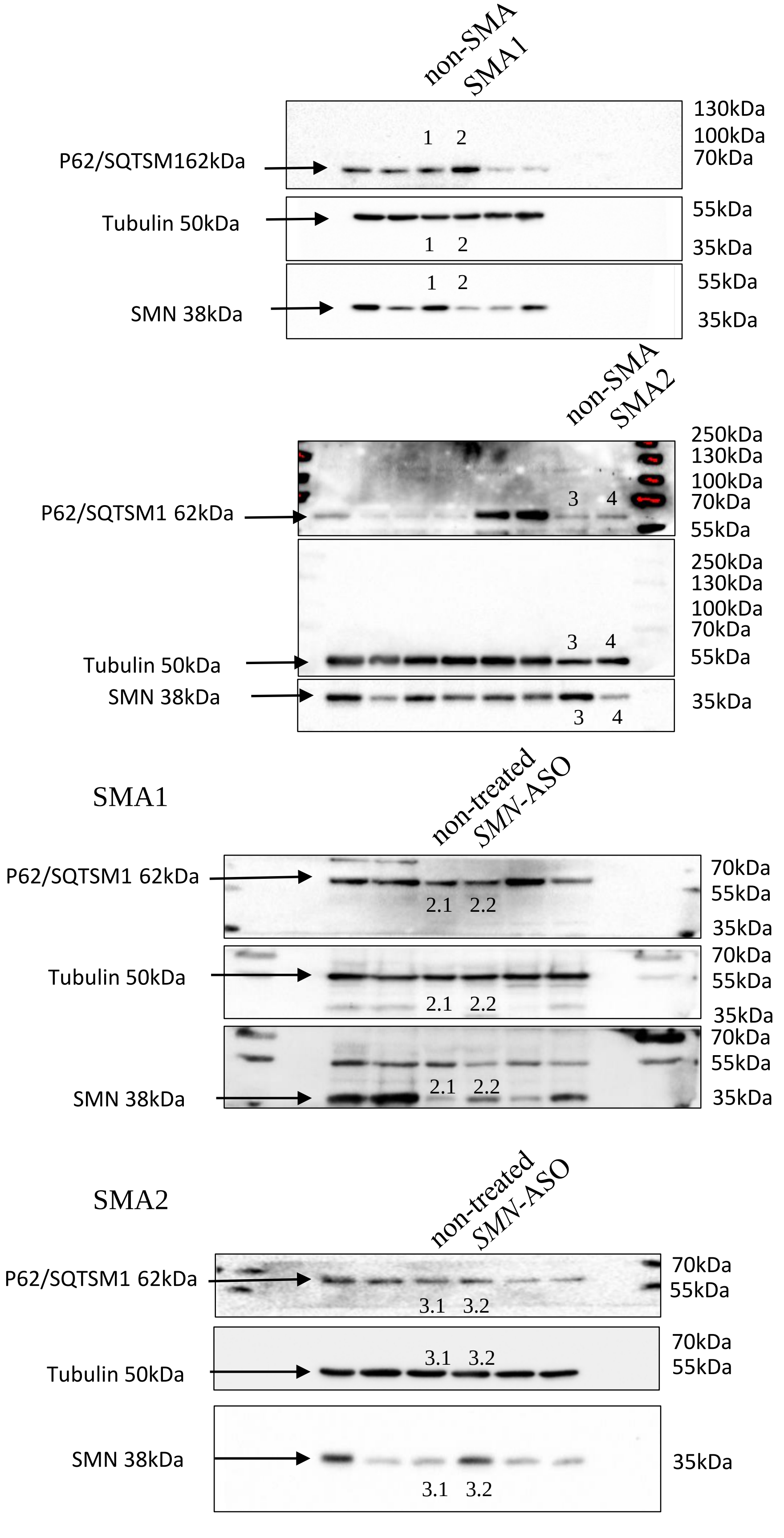

Full and uncropped western blot for Fig 4a, non-SMA Lane 1, SMA-carrier Lane 2, SMA1 Lane 3, and SMA2 Lane 4 are on the figure

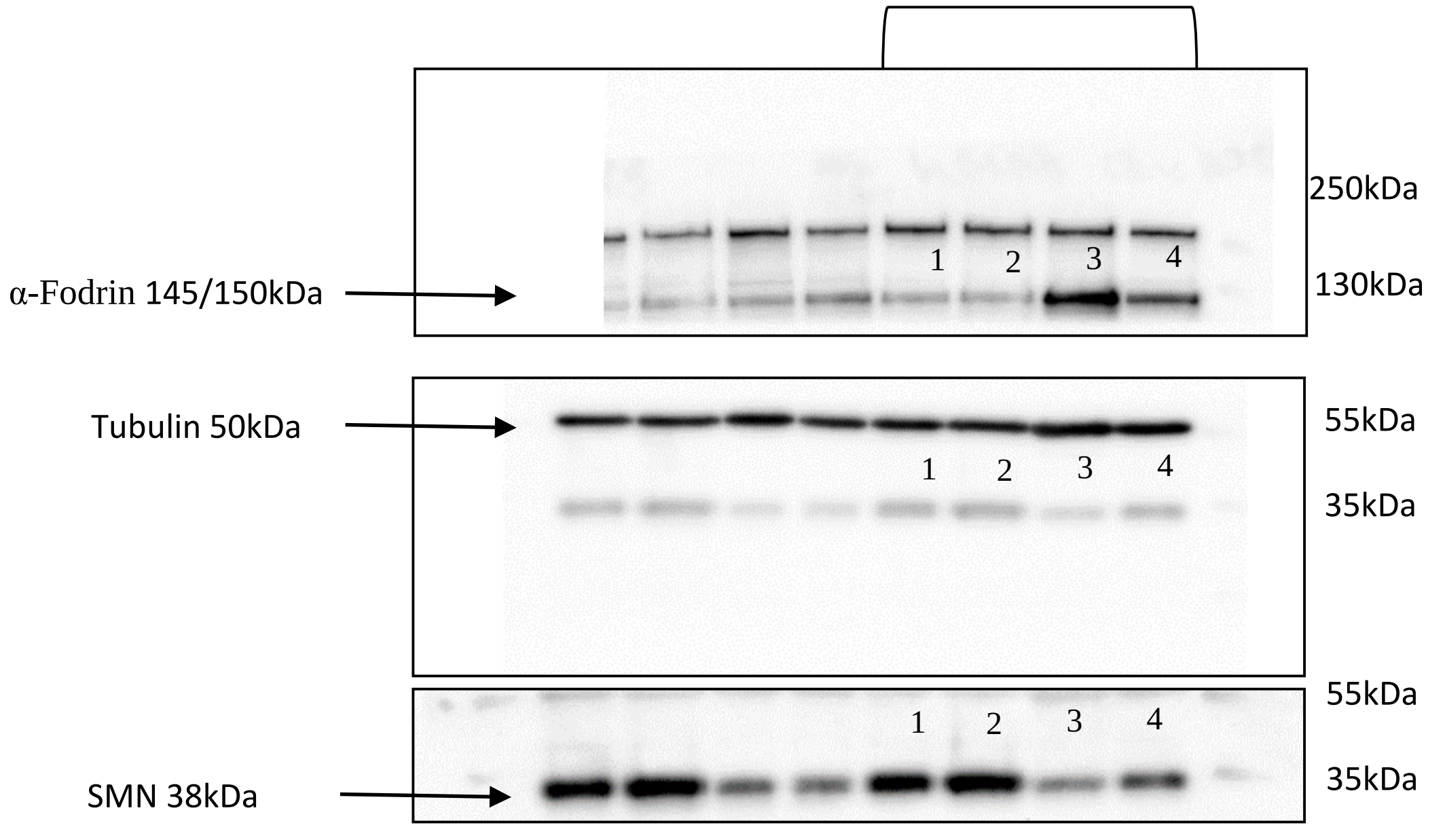

Full and uncropped western blots for Fig 4b, SMA1 non-treated Lane 1, SMA1 *SMN*-ASO Lane 2, SMA2 non-treated Lane 3, SMA2 *SMN*-ASO Lane 4 and Lane 4.1, SMA2 *SMN*-ASO+BAPTA Lane 4.2 are on the figures

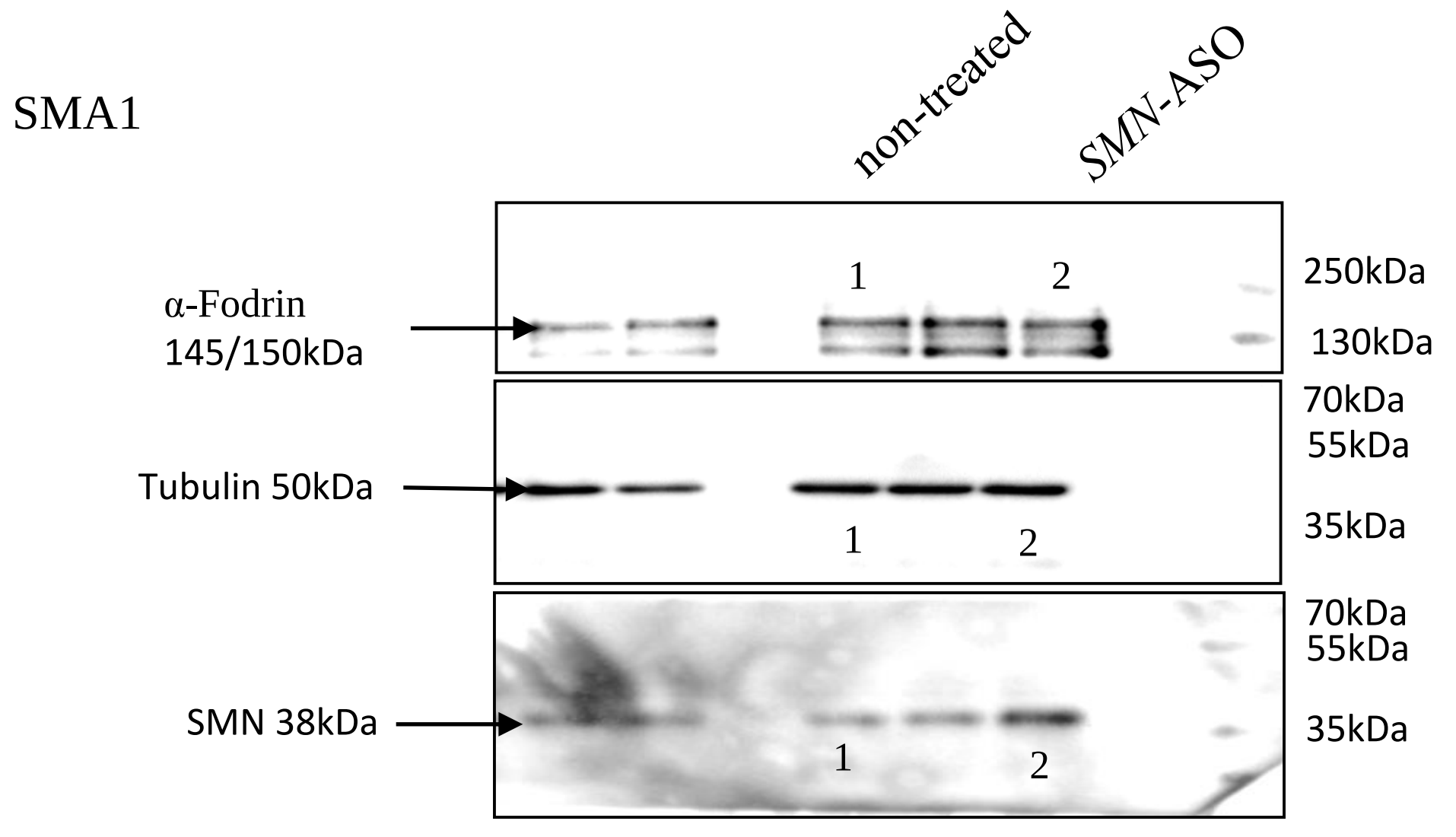

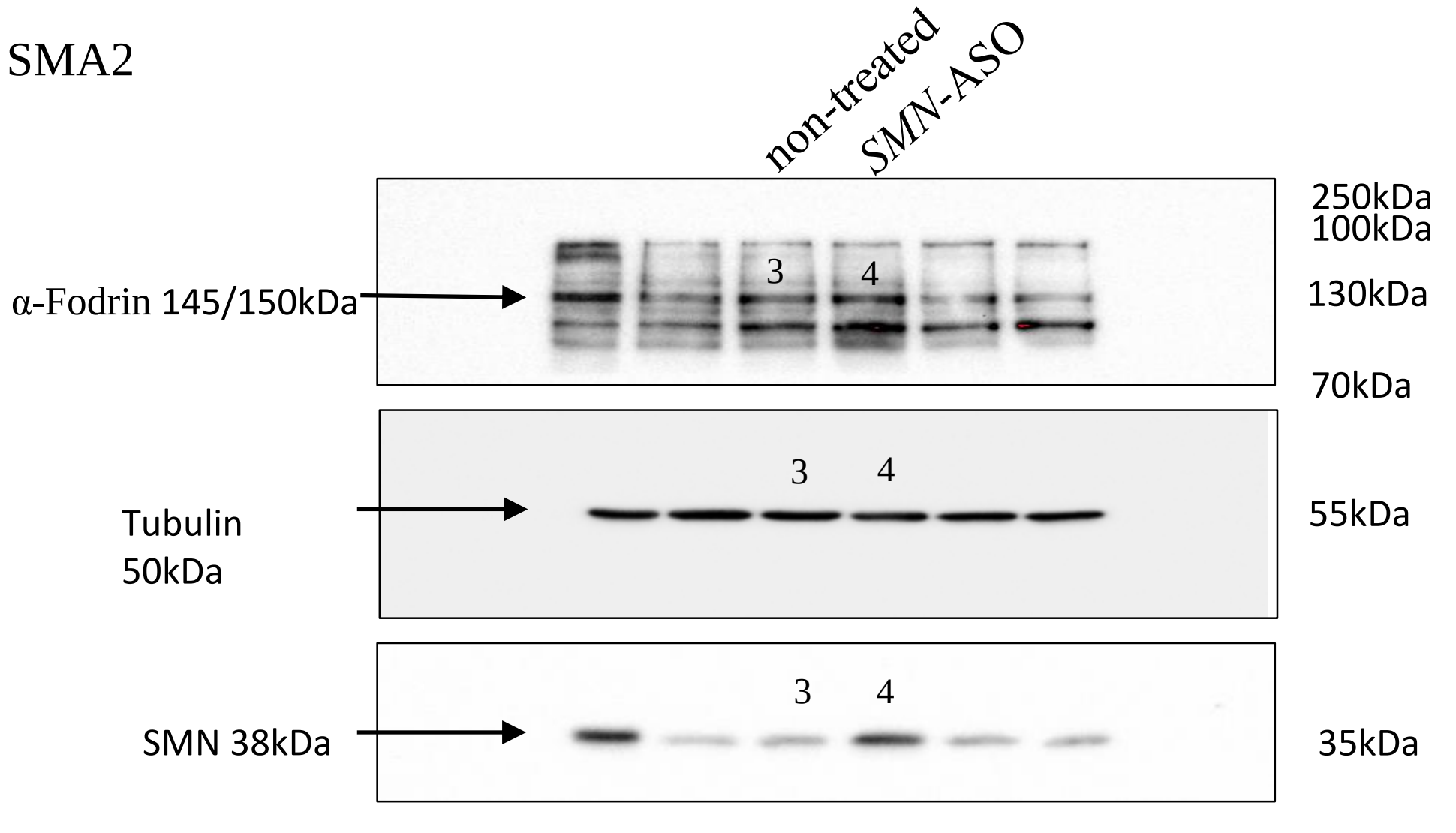

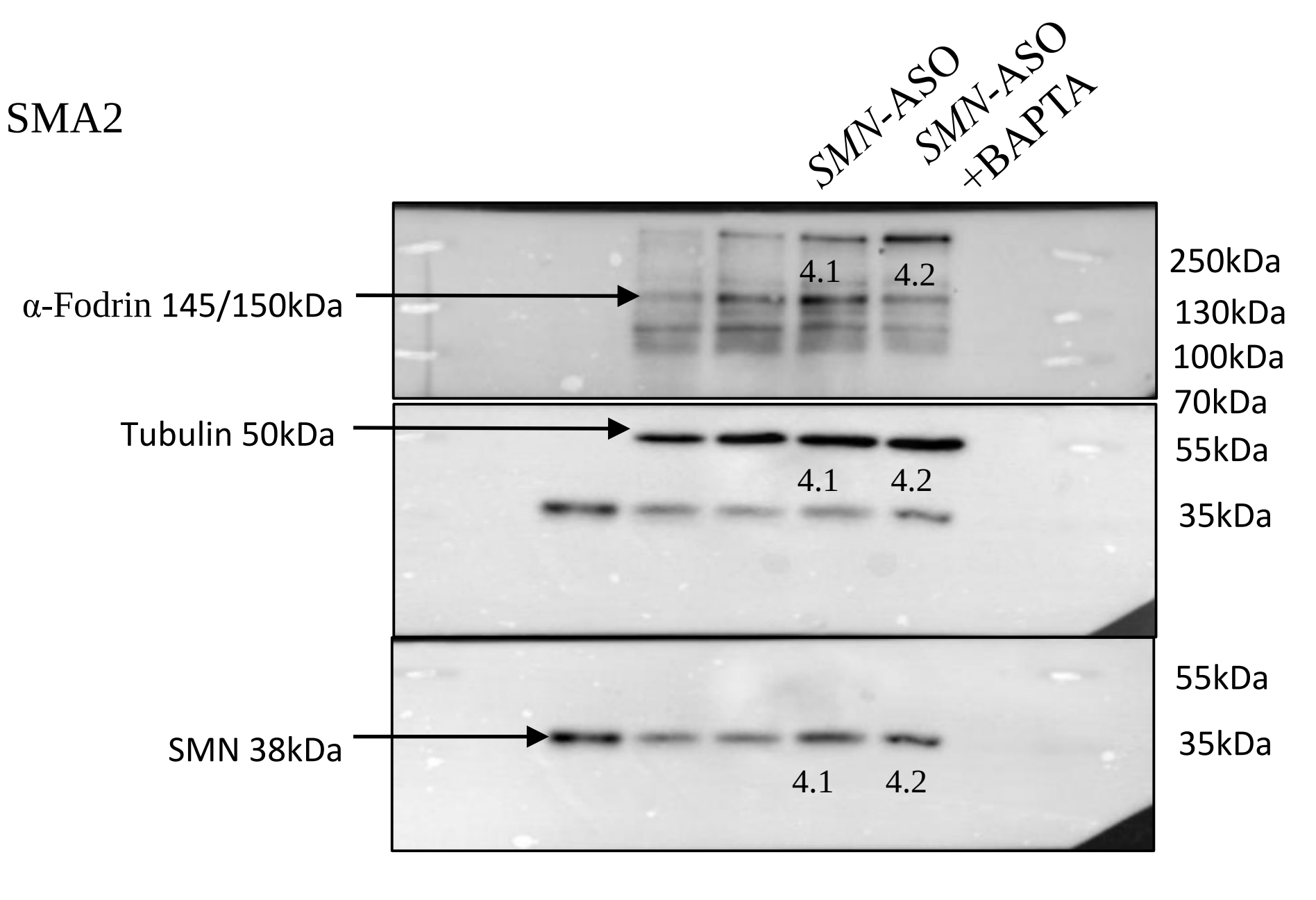

Full and uncropped western blot for Fig 4c, WT Lane 1, and SMNΔ7 Lane 2 are on the figure

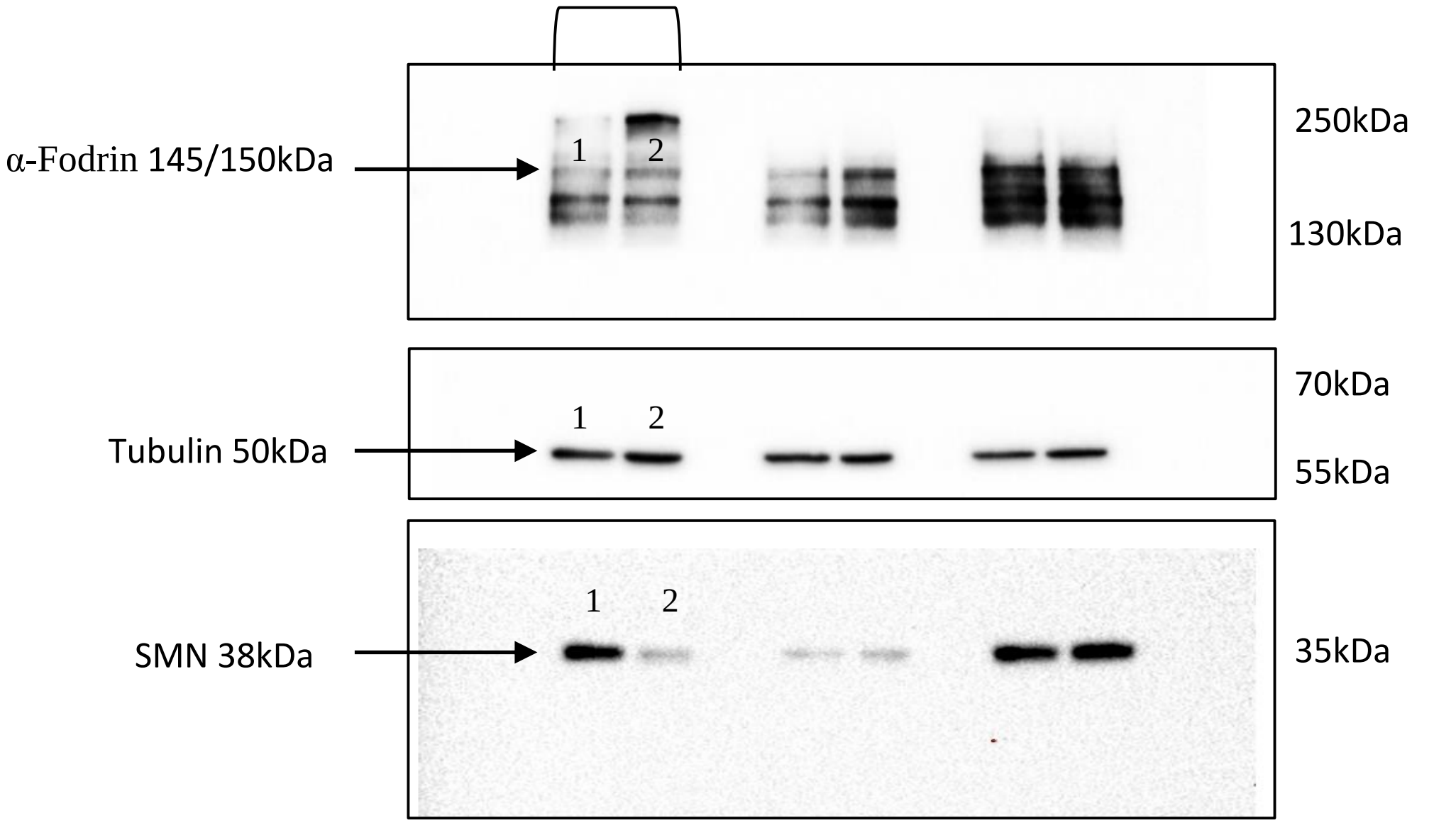

Full and uncropped western blot for Fig 4d, non-treated SMNΔ7 Lane 1, and *SMN*-ASO SMNΔ7 Lane 2 are on the figure

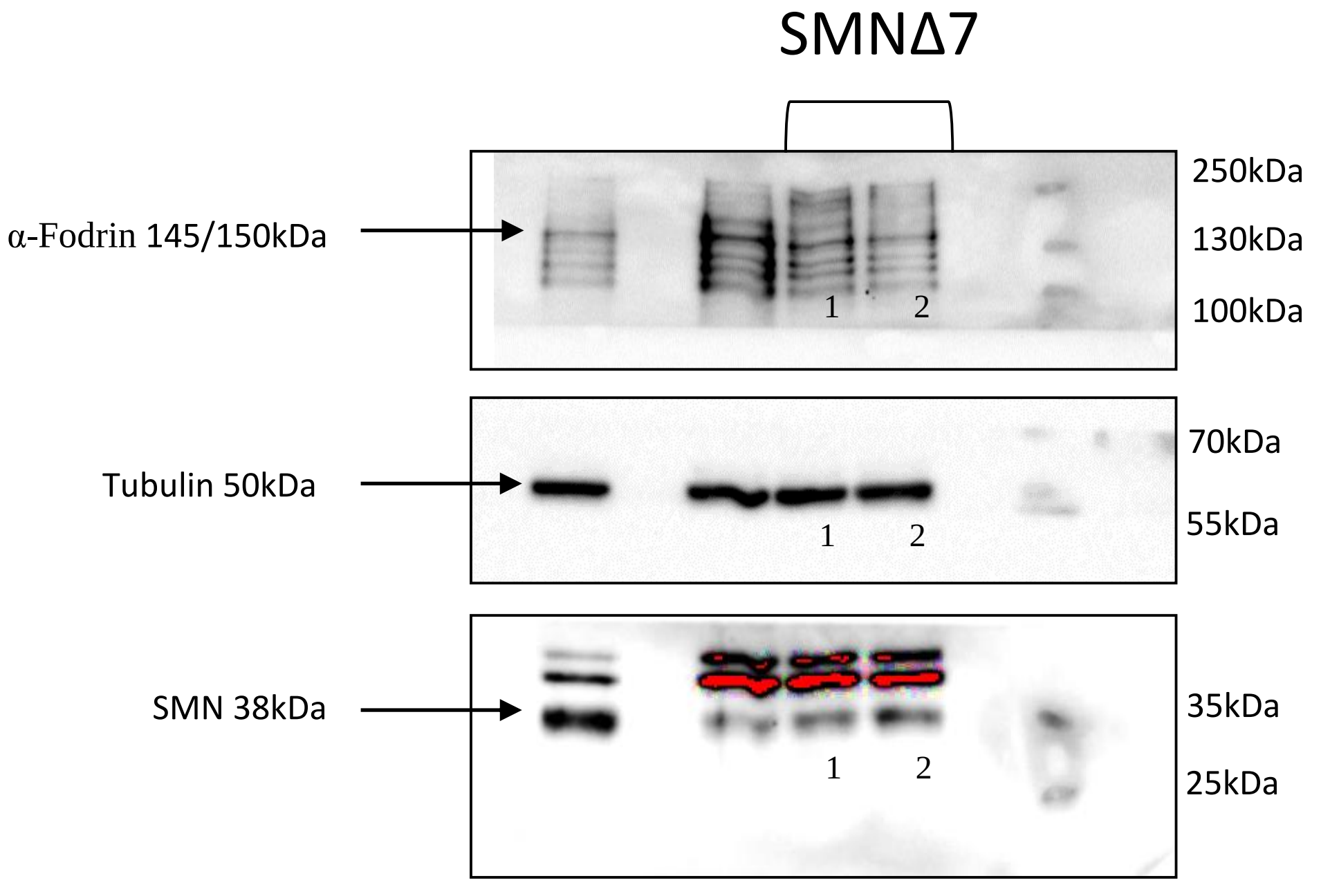

Full and uncropped western blot for Fig 5a, non-SMA Lane 1, SMA-carrier Lane 2, SMA1 Lane 3, and SMA2 Lane 4 are on the figure

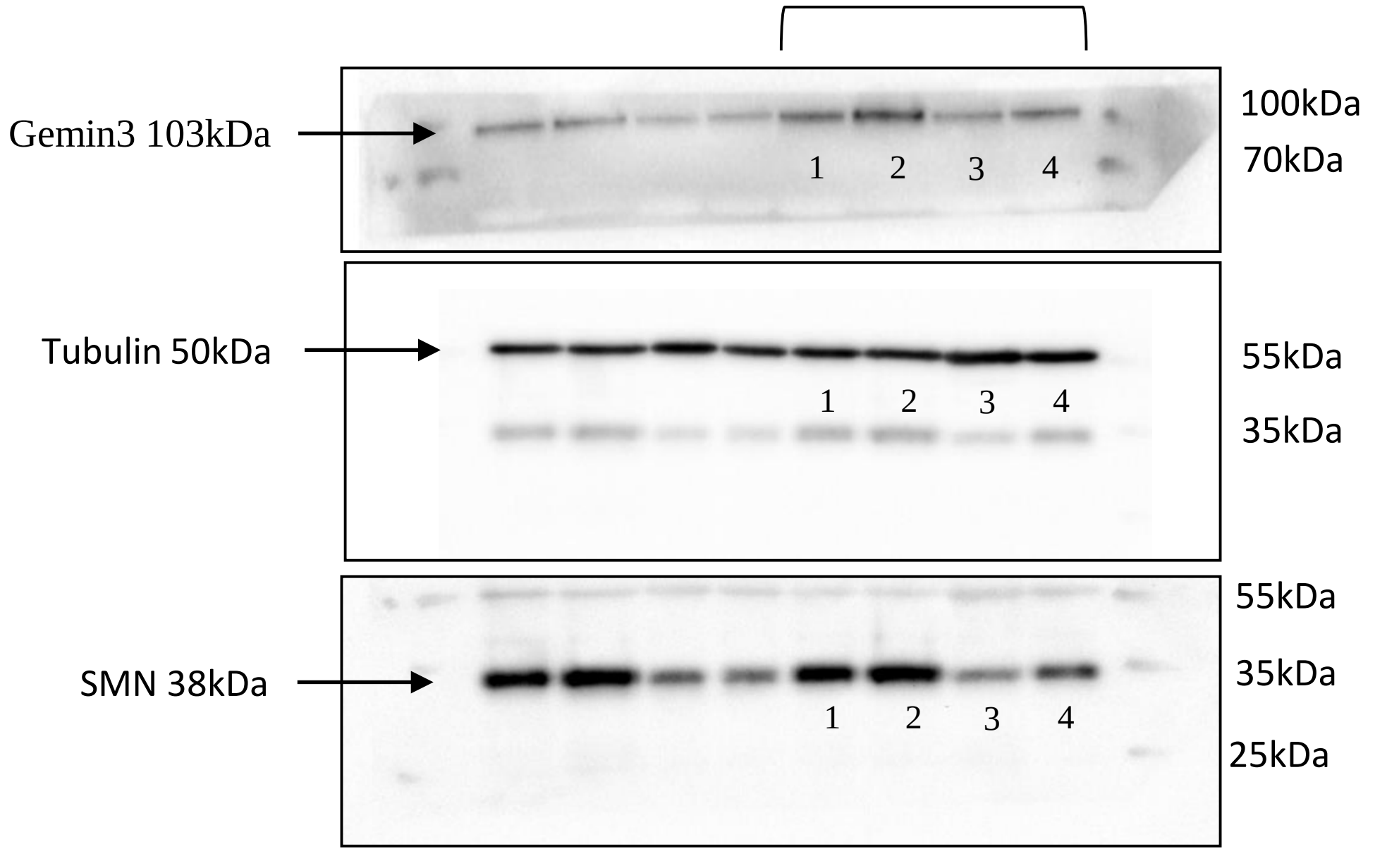

Full and uncropped western blots for Fig 5b (SMA1 top, SMA2 bottom), non-treated Lane 1, and *SMN*-ASO Lane 2 are on the figures

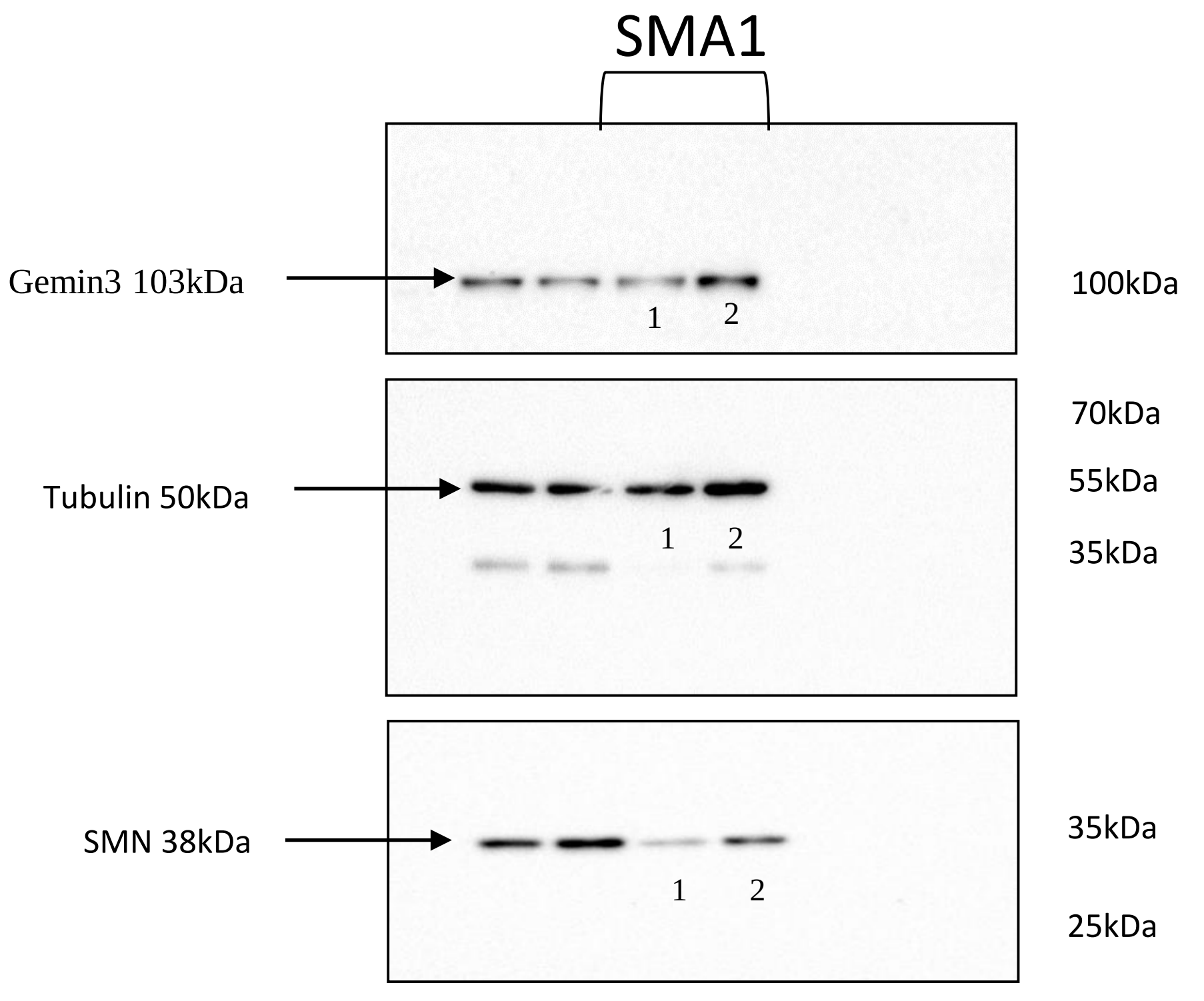

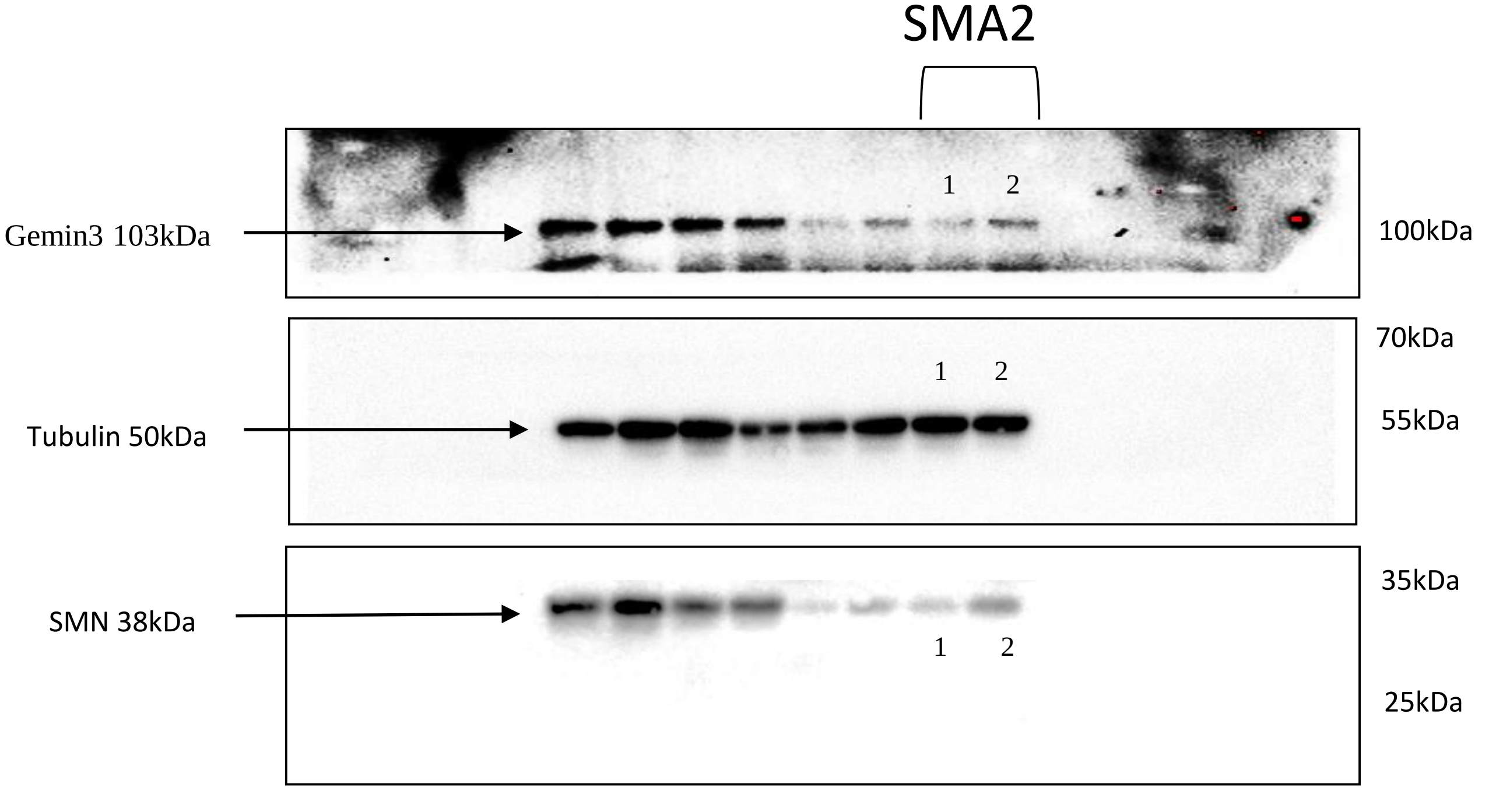

Full and uncropped western blot for Fig 5c, non-treated WT Lane 1, *SMN*-ASO WT Lane 2, non-treated SMNΔ7 Lane 3, and *SMN*-ASO SMNΔ7 Lane 4 are on the figure

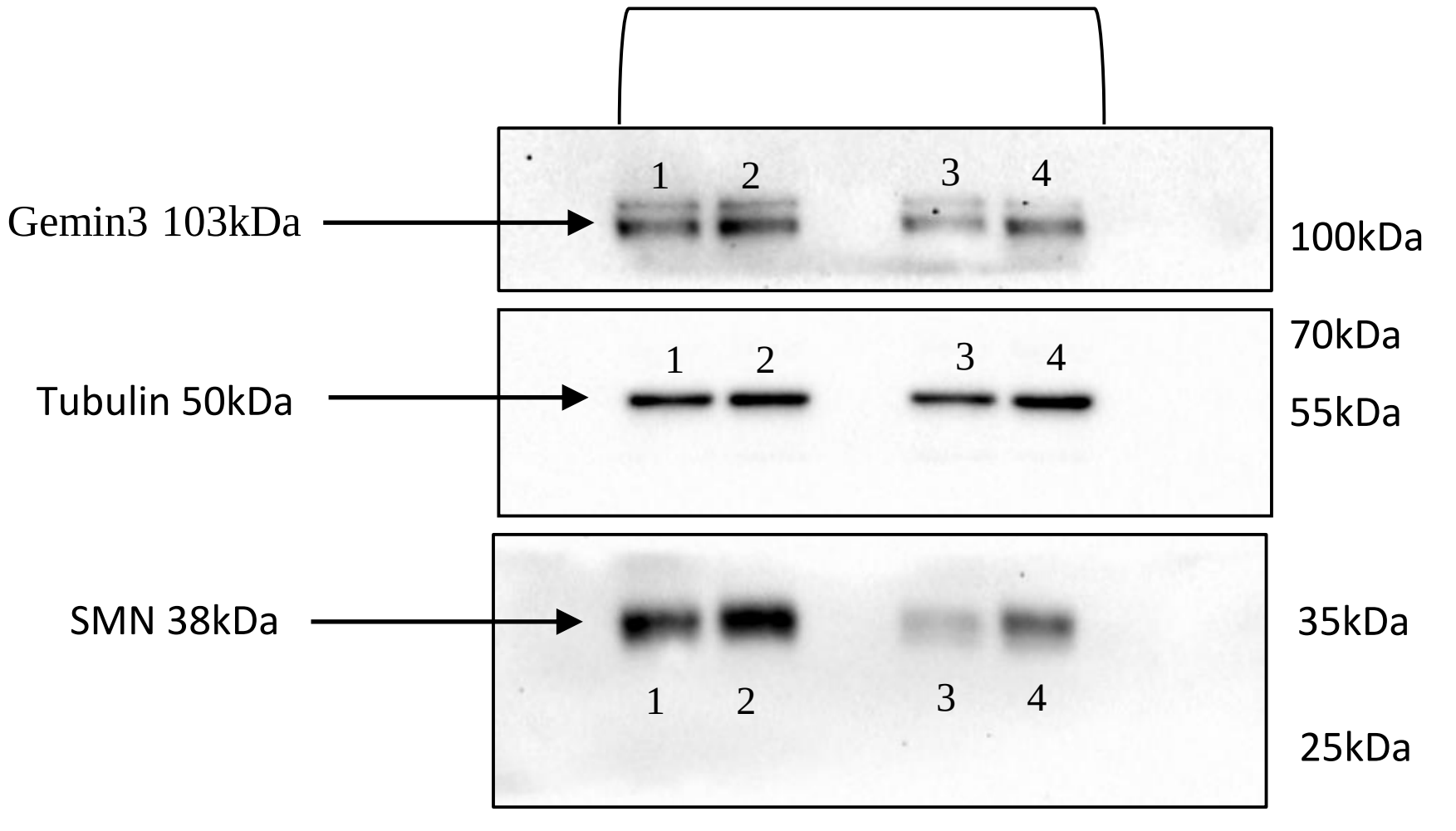

Full and uncropped western blot for Fig 6a (IKKβ top, RelA bottom), non-SMA Lane 1, SMA-carrier Lane 2, SMA1 Lane 3, and SMA2 Lane 4 are on the figure

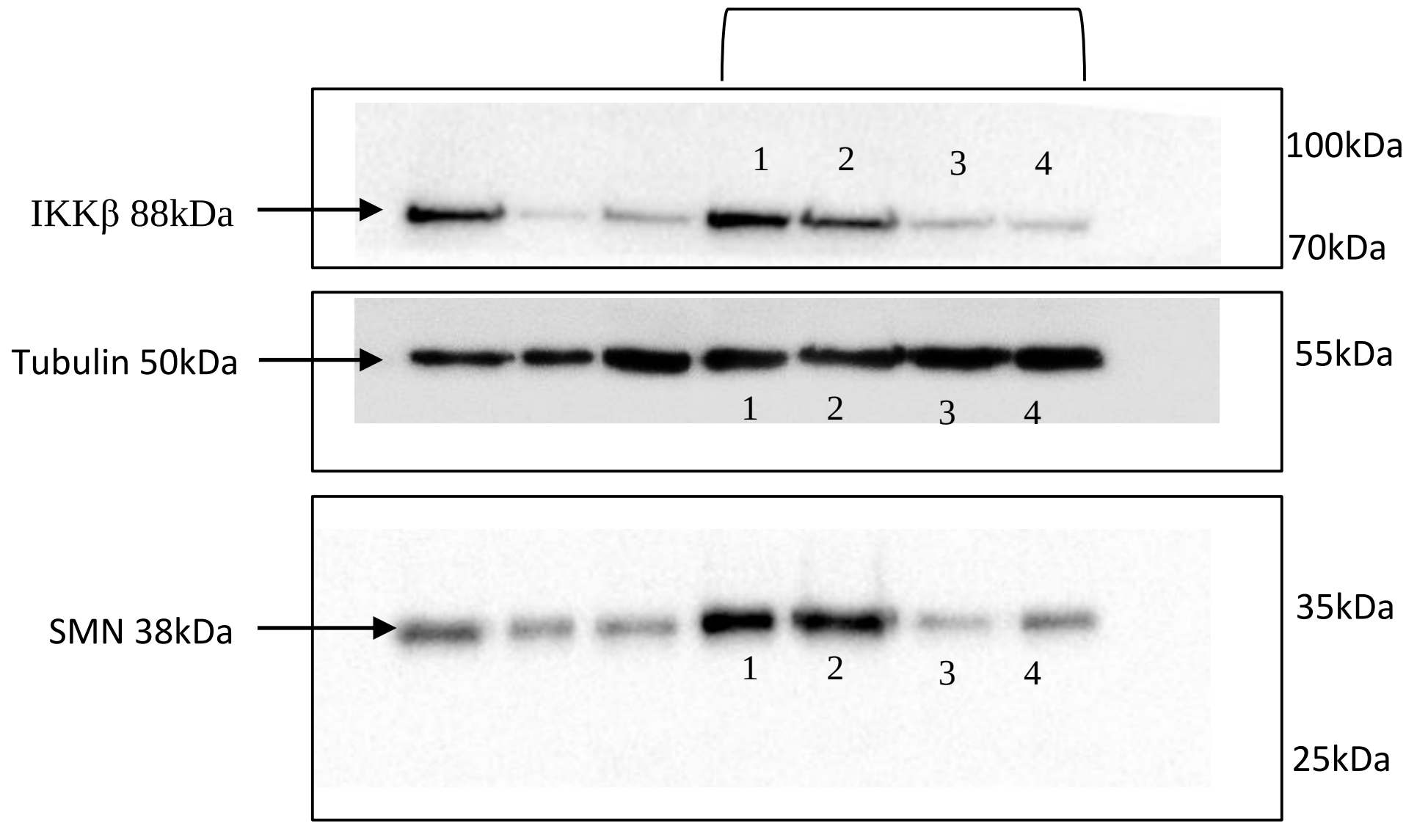

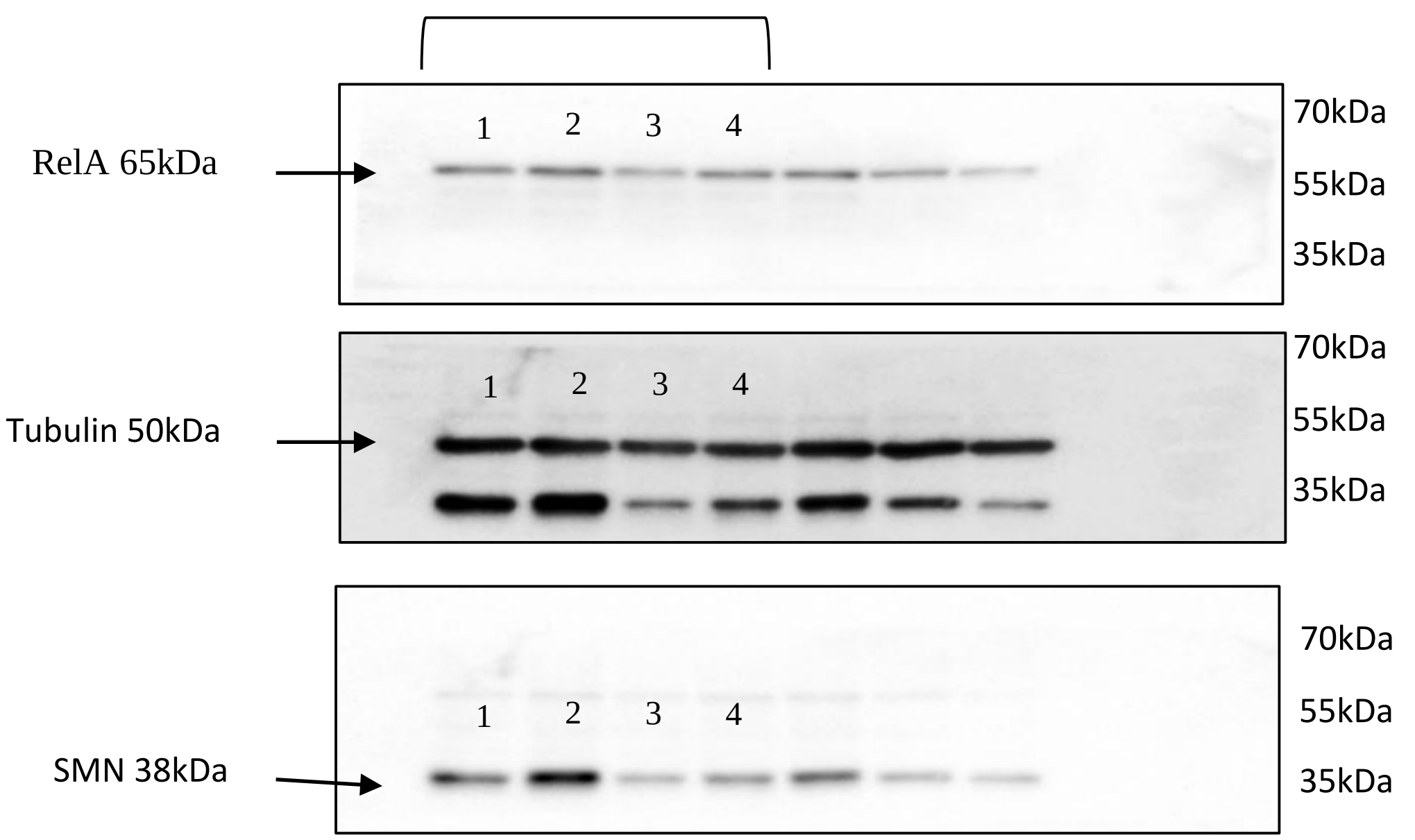

Full and uncropped western blots for Fig 6b left panel (IKKβ), non-treated Lane 1, and *SMN*-ASO Lane 2 are on the figures

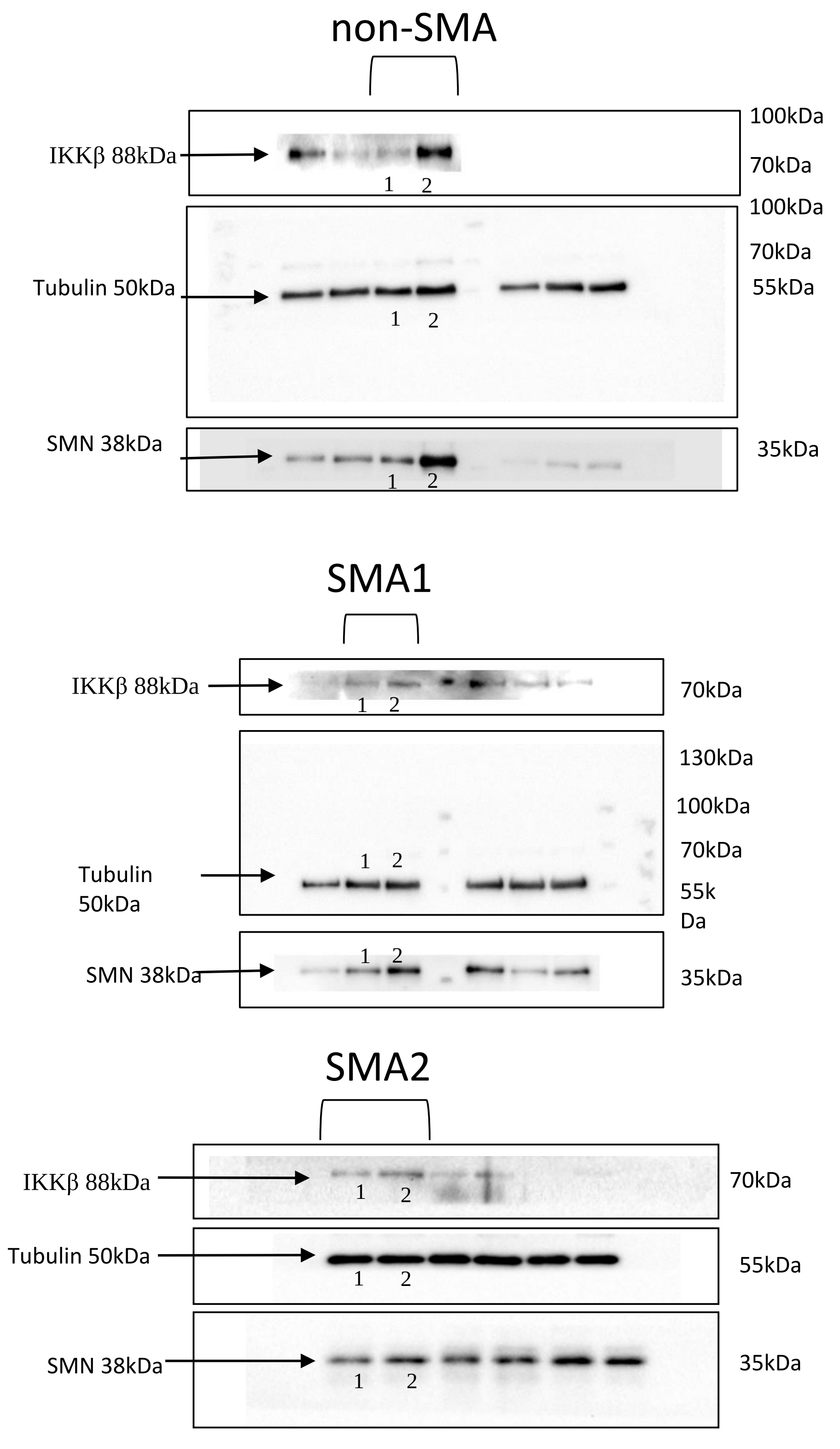

Full and uncropped western blots for Fig 6b right panel (RelA), non-treated Lane 1, and *SMN*-ASO Lane 2 are on the figures

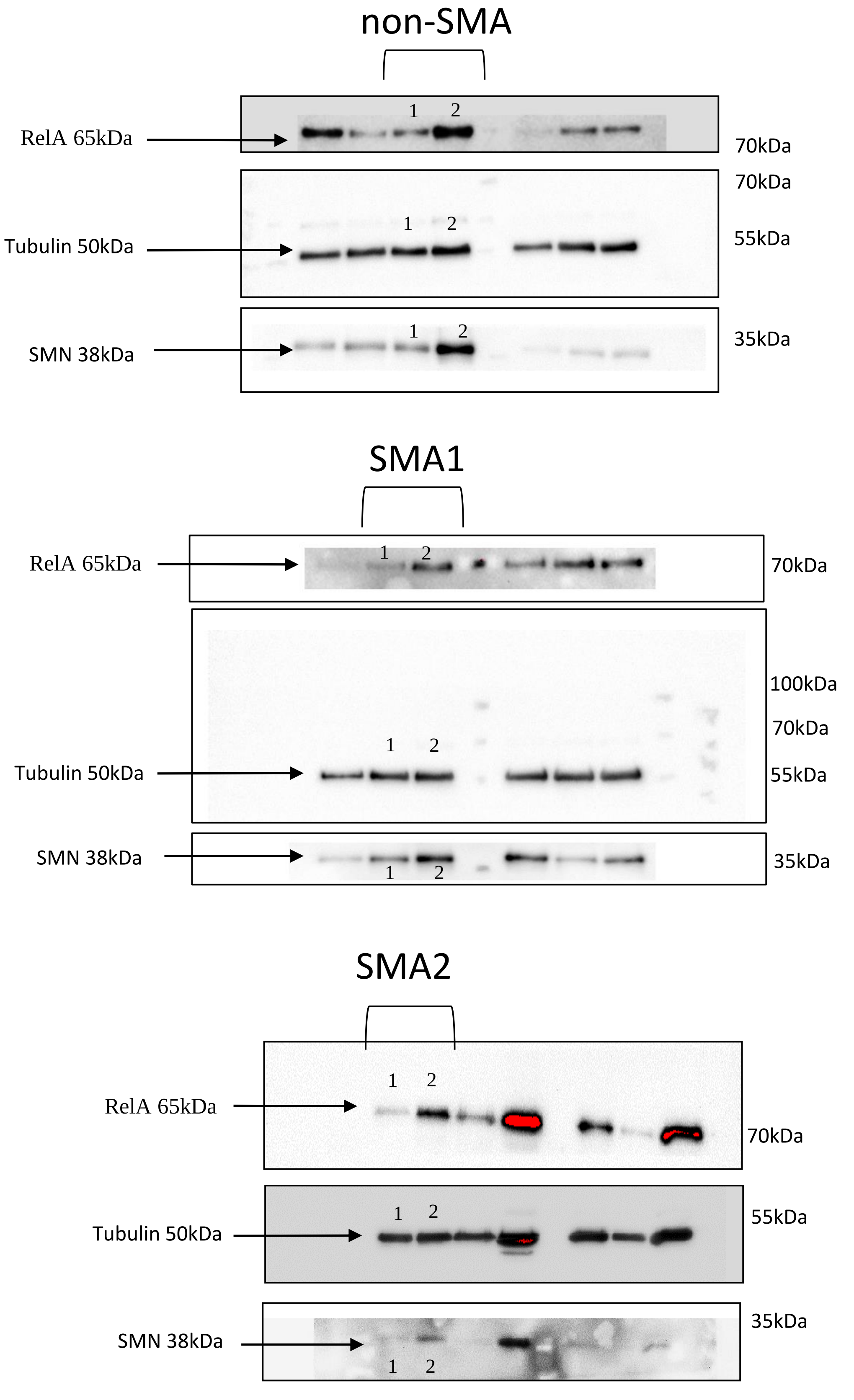

Full and uncropped western blots for Supplementary Fig 3 left panel (20 days), non-treated Lane 1, CTRL-ASO 60nM Lane 2, *SMN*-ASO 60nM Lane 3, CTRL-ASO 120nM Lane 4, and *SMN*-ASO 120nM Lane 5 are on the figures

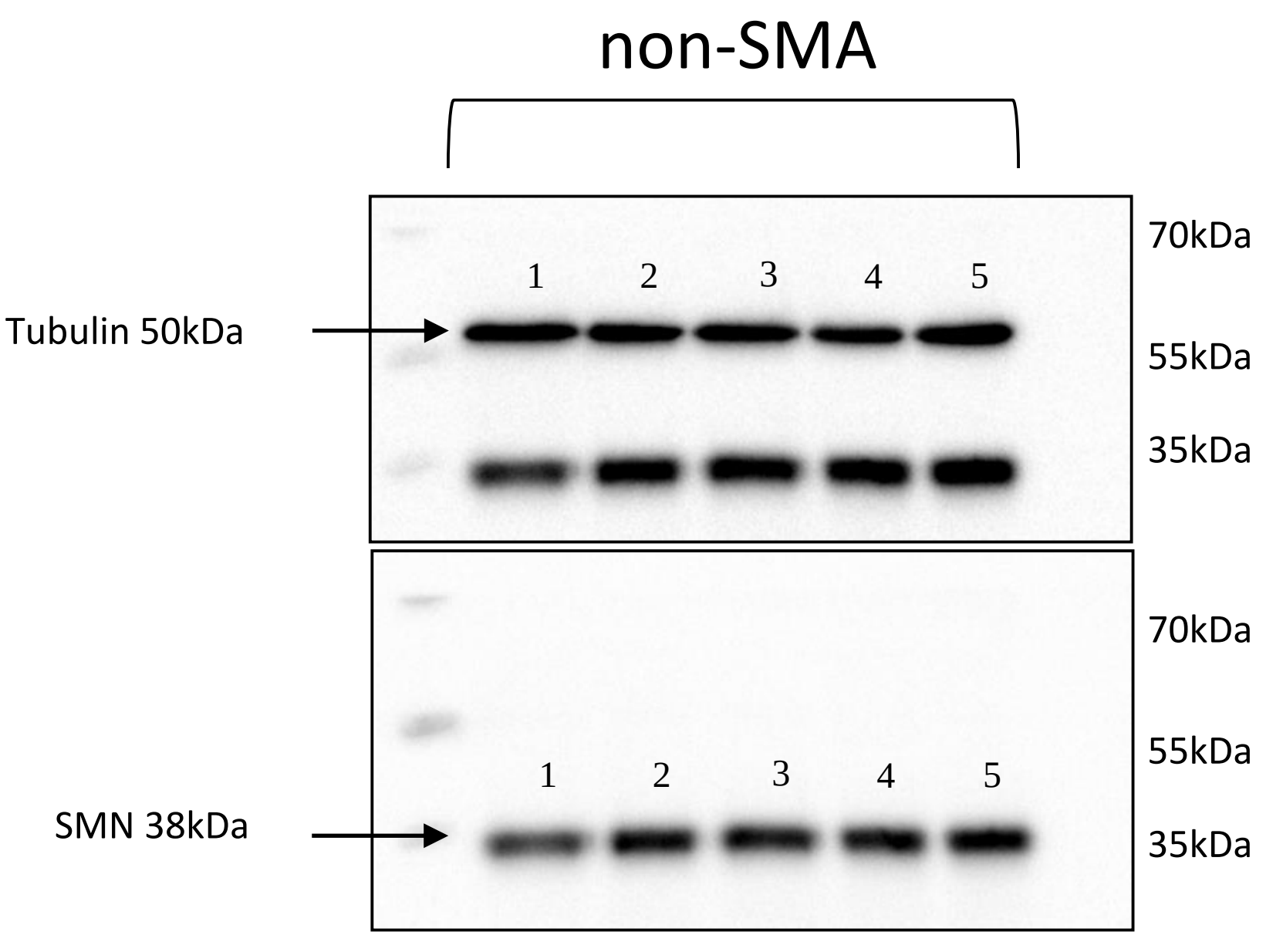

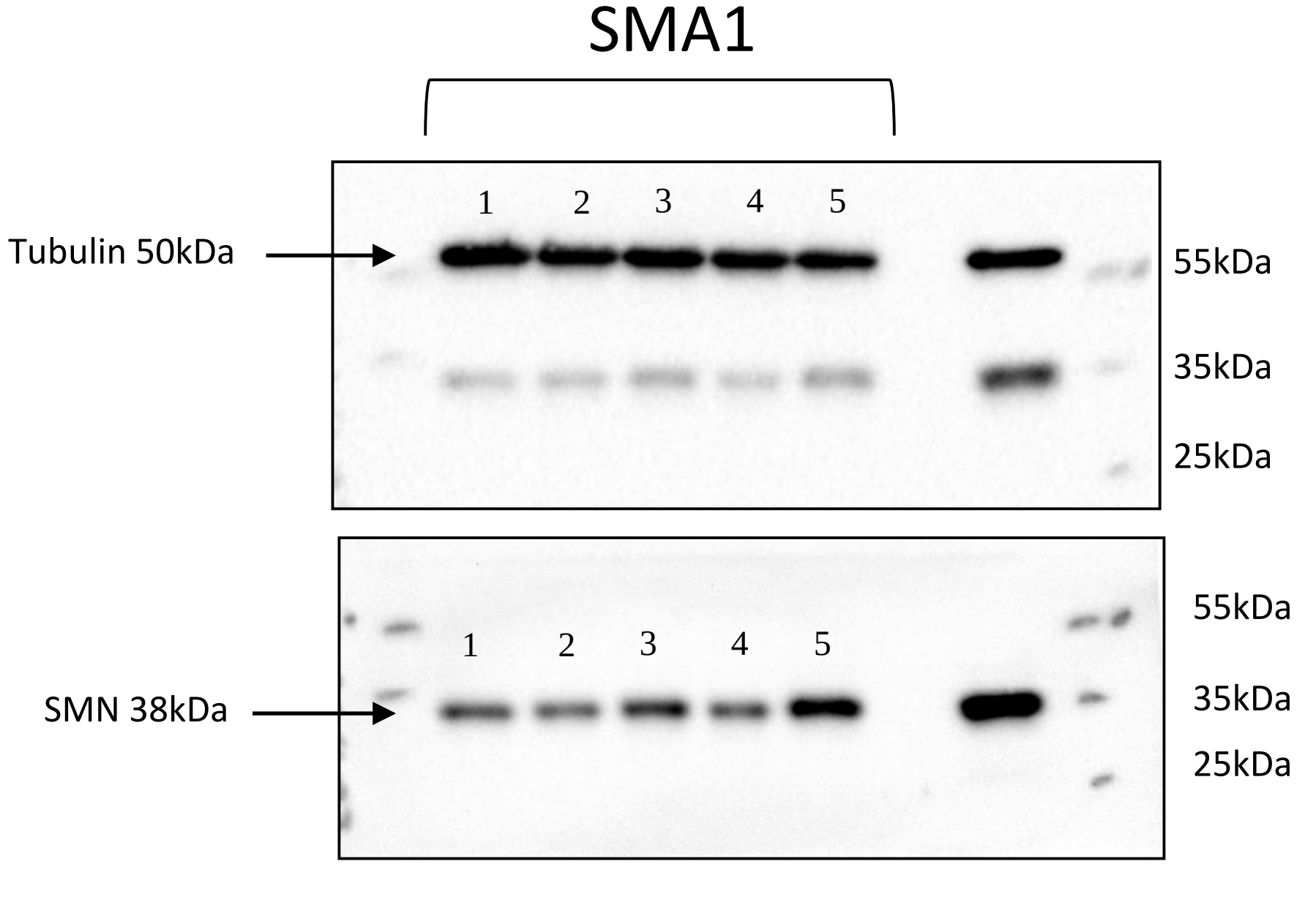

Full and uncropped western blots for Supplementary Fig 3 right panel (12 days), non-treated Lane 1, *SMN*-ASO 60nM Lane 2, *SMN*-ASO 120nM Lane 3, *SMN*-ASO 250nM Lane 4, *SMN*-ASO 500nM and *SMN*-ASO 1μM Lane 5 are on the figures

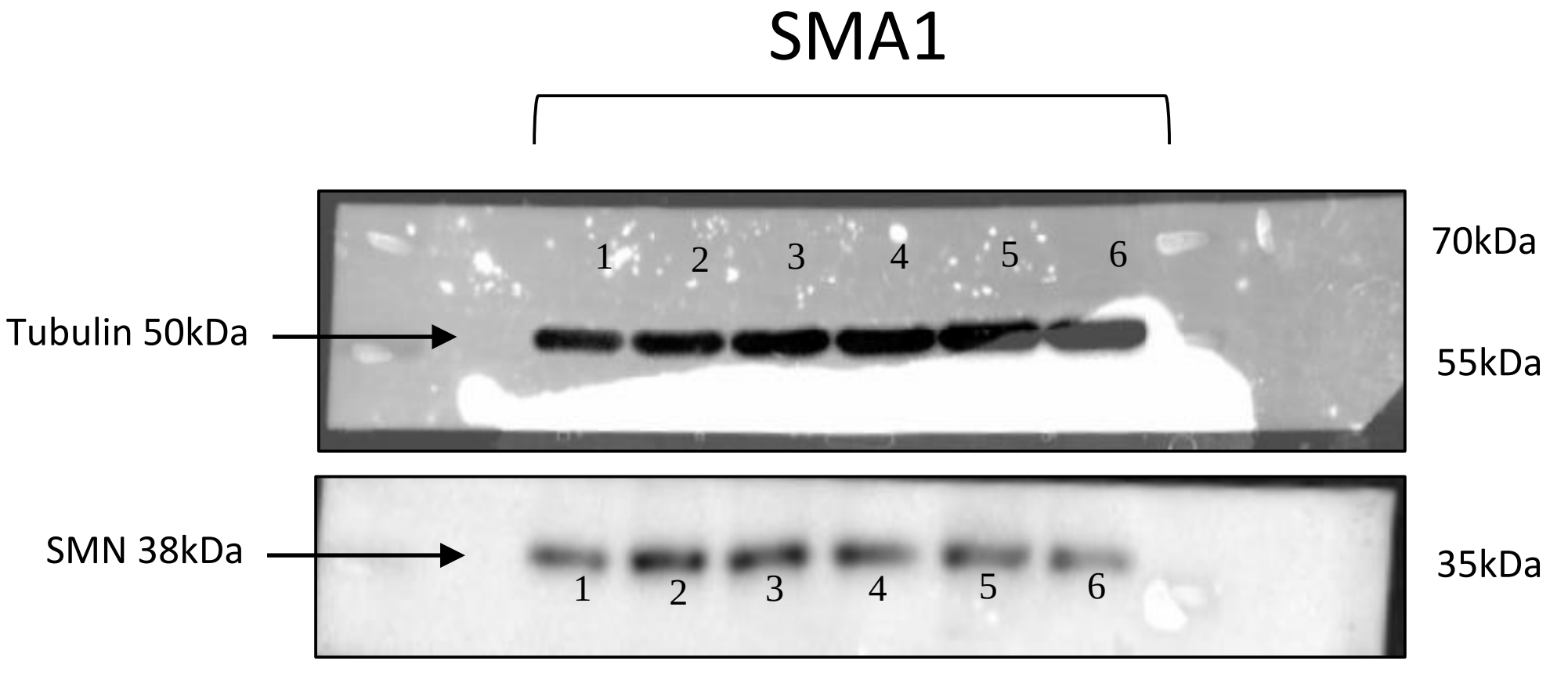

Supplement: Supplementary file 2 [file 41419_2026_9105_MOESM2_ESM.pdf]
